# Procyanidins: Structural Properties, Production Methods, and Modern Applications

**DOI:** 10.3390/molecules31020223

**Published:** 2026-01-08

**Authors:** Aleksandr Yu. Zakharov, Dmitriy Berillo, Annie Ng, Damir S. Aidarkhanov, Anna V. Tukesheva, Kamila M. Temirkulova, Ainur Tanybayeva, Zulkhair A. Mansurov, Mannix P. Balanay, Vladimir V. Pavlenko

**Affiliations:** 1 The Institute of Combustion Problems, St. Bogenbai Batyr, 172, Almaty 0500012, Kazakhstan; 2 Nazarbayev University Research Administration, Kabanbay Batyr Ave., 53, Astana 010000, Kazakhstanaidarkhanov@nu.edu.kz (D.S.A.); 3 International Campus, M. Kozybayev North-Kazakhstan University, Pushkin Street, 86, Petropavlovsk 150000, Kazakhstan; 4 Department of Electrical and Computer Engineering, Nazarbayev University, Kabanbay Batyr Ave., 53, Astana 010000, Kazakhstan; 5 Department of Geography and Environmental Sciences, Al-Farabi Kazakh National University, al-Farabi Ave., 71, Almaty 050040, Kazakhstan; 6 Department of Chemistry, Al-Farabi Kazakh National University, al-Farabi Ave., 71, Almaty 050040, Kazakhstan; 7 Institute of Functional Materials, Nazarbayev Ave., 43, Almaty 050016, Kazakhstan; 8 Department of Chemistry, Nazarbayev University, Kabanbay Batyr Ave., 53, Astana 010000, Kazakhstan; mannix.balanay@nu.edu.kz

**Keywords:** procyanidins, review, extraction methods, synthetic methods, procyanidins conformations

## Abstract

Procyanidins, a class of substances widely distributed in nature, have attracted the attention of the scientific community due to their bioactive properties, especially with regard to human health. This review is based on an extensive examination of peer-reviewed literature, patents, and clinical trial reports published between 2005 and 2025. From an initial pool of more than 300 documents, 283 studies were selected according to criteria of scientific rigor, methodological clarity, and relevance to the research objectives. A literature search was performed using PubMed, PubChem, Google Scholar, Scopus and ResearchGate employing keywords such as Procyanidins, chemical structure, extraction, and health effects. This article provides a comprehensive overview of current methods for obtaining these compounds, which include both natural sources and synthetic approaches. It provides a concise summary of the molecular structure of procyanidins and emphasizes the importance of understanding their conformational features for predicting biological activity. The challenges of establishing correlations between the structural features of procyanidins and their properties are described. In addition, this article explores the many potential applications of these compounds, spanning both biochemistry and the field of design and synthesis of novel materials. This review provides a comprehensive evaluation of Procyanidins, focusing on their geometrical conformation analysis through advanced NMR spectroscopy techniques including homonuclear correlation (COSY, TOCSY), heteronuclear one-bond (HSQC, HMQC), multiple-bond (HMBC) experiments, and through-space correlation (NOESY) in conjunction with various extraction methodologies.

## 1. Introduction

Polyphenolic compounds represent one of the largest and most widespread groups of natural substances, attracting considerable attention due to their broad spectrum of biological activity and beneficial properties [1,2,3]. These compounds are abundant in various plants [4,5], fruits [6,7], wines [8,9,10], apples [11], beans [12], cacao [13,14] and many other sources. They can be isolated from pine bark of several species, Siberian fir bark, blueberry leaves, and other plant materials [15,16,17,18].

Within this diverse group, particular attention is given to procyanidins, which represent the most commonly consumed class of bioflavonoids in the human diet. These compounds were first described in 1948 with successfully isolated and characterized the phytonutrient now known as oligomeric procyanidins (OPCs). That same year, Masquelier filed a patent for the first industrial method to produce OPC-based products. He later patented techniques for isolating these compounds from pine bark in 1951 and from grape seeds in 1970 [19].

**Figure 1:**
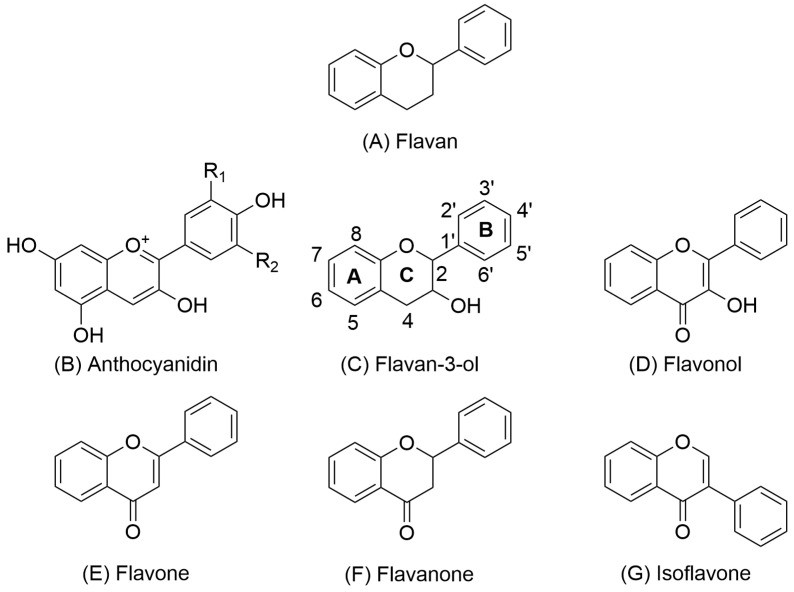
Representative chemical structures of key flavonoid subclasses, including flavan (**A**), anthocyanidin (**B**), flavan-3-ol (**C**), flavonol (**D**), flavone (**E**), flavanone (**F**), and isoflavone (**G**). Structural variations in the oxidation state and hydroxylation pattern of the central C ring define the chemical and biological properties of each subclass.

Procyanidins are a subclass of proanthocyanidins (well known as condensed tannins) [15,20,21]. Proanthocyanidins are characterized by high molecular weight and consist of oligomeric or monomeric subunits of flavan-3-ols. Flavan-3-ols include 2 phenolic rings A and B and a heterocyclic ring C (Figure 1) [8,22].

The diversity of procyanidins arises from variations in the type and position of functional groups attached to the A and B rings of their flavan-3-ol subunits. In the case of procyanidins, catechin and epicatechin serve as the primary monomeric building blocks [23,24]. Some authors include compounds containing structurally related gallocatechin and epigallocatechin units within the procyanidin family. However, these are more commonly classified as a separate group known as prodelfinidins [25,26].

Procyanidins are categorized according to their degree of polymerization oligomers consist of 2–10 monomeric units, while polymers contain more than 10. As the degree of polymerization increases particularly beyond tetramers the number of known procyanidin structures decreases significantly [27]. Although OPCs are of great interest due to their diverse biological activities, their chemical synthesis remains challenging and cost-intensive [28]. Therefore, the following discussion focuses primarily on naturally derived compounds, particularly dimeric procyanidins.

**Figure 2:**
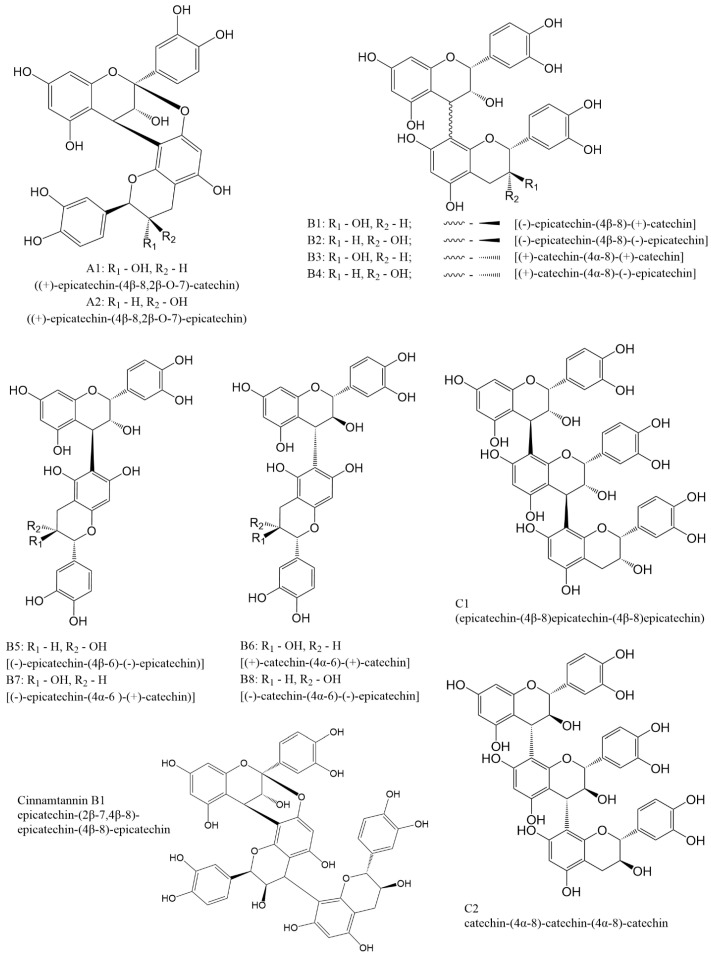
Structures of most common procyanidins.

In dimeric procyanidins, catechin and epicatechin units are linked through carbon atoms C4, C6, or C8. Depending on the specific linkage site and the degree of polymerization, procyanidins are divided into three main types A, B, and C. Types A and B correspond to dimeric forms, whereas type C includes trimeric structures (Figure 2).

Group A is characterized by the presence of carbon C4-C8 and ether C2-O-C7 bonds and has only two representatives A1 and A2. Group B includes compounds formed by C4-C8 or C4-C6 carbon bonds. This type of procyanidins has 8 representatives B1 [(−)-epicatechin-(4β-8)-(+)-catechin], B2 [(−)-epicatechin-(4β-8)-(−)-epicatechin], B3 [(+)-catechin-(4α-8)-(+)-catechin], B4 [(+)-catechin-(4α-8)-(−)-epicatechin], B5 [(−)-epicatechin-(4β-6)-(−)-epicatechin)], B6 [(+)-catechin-(4α-6)-(+)-catechin], B7 [(−)-epicatechin-(4α-6)-(+)-catechin)] and B8 [(−)-catechin-(4α-6)-(−)-epicatechin]. Procyanidins of the C group are formed by C4β-C8 bonds between catechin blocks (procyanidin C1) or by C4α-C8 bonds between epicatechin blocks (procyanidin C2).

Besides the compounds mentioned above, there are several procyanidins that do not have specific names ent-catechin-(C2α-O7, C4α-C8)-catechin, ent-epicatechin-(C2α-O7, C4α-C8)-catechin, (−)-epicatechin-(C4β-C8)-(+)-catechin, epicatechin(C4β-C8)-epicatechin(C4β-C8)-catechin, and epicatechin(C4β-C8)-catechin(C4α-C8)-epicatechin. A number of trimers with A-type bonds or mixed A- and B-type bonds are known, some of which are a major constituent of a cinnamon [29,30]. Most of these compounds can be obtained either through extraction procedures or via the synthetic routes discussed later in this review.

Procyanidins are of great interest in modern medicine because of their diverse biological activities, including antioxidant, antiarrhythmic, and hypotensive effects [5,31,32]. They have demonstrated anticancer potential [33,34,35], exhibit anti-inflammatory properties [34,36], and possess anti-aging activity [37,38]. Since their bioactivity has been extensively reviewed elsewhere [15,28,39,40,41,42,43], it will not be addressed in detail here.

Beyond their biological effects, the structural features of procyanidins enable their use in a range of technological applications. The presence of adjacent hydroxyl groups in the 4′ and 5′ positions of the B-ring allows their use as chelating agents for heavy metal sorption or as components in drug delivery systems (Figure 1). Common carrier materials include iron oxide [44], gold [45,46], silver [46,47], and chitosan nanoparticles [48]. Additionally, the multiple hydroxyl groups at opposite ends of the molecule make procyanidins excellent linkers in hydrogel networks, particularly in tissue engineering [49,50] and wound dressing applications [51]. A notable advantage of such materials is their high biocompatibility.

## 2. Structural Properties

**Figure 3:**
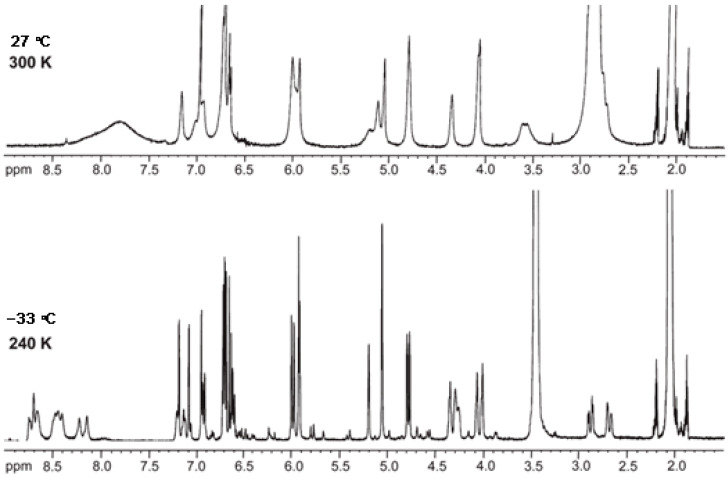
Effect of lowering the temperature from 27 °C to −33 °C on the ^1^D ^1^H NMR spectrum of procyanidin C1 in acetone-d_6_ [62].

Understanding structural features of individual procyanidins in solution can be the key to solving more complex problems that are crucial for understanding their bioactivity, as it influences molecular interactions with biological targets. Although the bioactivities of procyanidins are well characterized, the mechanisms responsible for them are largely unexplored [10,52]. Several structural aspects of these compounds can be highlighted:

- Molecules of procyanidins have several chiral centers, which dictate stereospecific binding and enzymatic recognition, affecting bioavailability and stability;
- Heterocyclic ring C of flavanol subunits can adopt different conformations (armchair, semi-chair, boat), altering molecular flexibility and solubility in aqueous environments;
- Ring B can occupy either an axial or an equatorial position relative to ring C, impacting intermolecular hydrogen bonding and self-association tendencies;
- Procyanidin types B and C can have several rotamer forms around interflavan bonds, influencing aggregate formation and precipitation behavior (Figure 3).

These features affect the bioavailability of procyanidins, their stability, self-association mechanisms, and processes of colloid and precipitate formation [52,53,54].

Nuclear magnetic resonance spectroscopy (NMR) [55], high-performance liquid chromatography-mass spectrometry (HPLC-MS) [56], matrix-assisted laser desorption ionization (MALDI) [57,58], and computational methods of molecular mechanics [59] and quantum-chemical modeling using the density functional theory (DFT) [54] are commonly used as methods to investigate and confirm the structure. It should be noted that DFT modeling of procyanidins shows the strong dependence of the calculated properties on the chosen functional, basis set, and solvation model. For small procyanidins, the use of M05-2X and M06-2X functionals with medium-sized basis sets provides more accurate descriptions of intramolecular hydrogen bonding and π–π interactions compared to the commonly employed B3LYP functional. For instance, the calculated bond dissociation enthalpies of the B1 dimer vary significantly depending on the computational assumptions, which can directly influence the predicted preference for the HAT antioxidant mechanism over SET-PT [60].

NMR spectroscopy methods include homonuclear correlation (COSY, TOCSY), heteronuclear one-bond (HSQC, HMQC) and multiple-bond (HMBC) experiments and through-space correlation method (NOESY) [59,61]. The challenges in NMR analysis of procyanidins include isomer separation due to interflavan bond rotation (e.g., in B1-B4) and dynamic heterocyclic ring conformational changes, which can be mitigated by acylating hydroxyl groups or recording spectra at lower temperatures (Figure 3) [24,62]. However, the final challenge lies in determining the absolute configuration of the constituent monomeric units. While the H-2/H-3 relative configuration can be readily established using coupling constants, resolving the H-3/H-4 configuration becomes difficult in H-2/H-3 cis-isomers. Moreover, the use of NOESY correlations is limited by spin diffusion caused by the increased rotational flexibility of these molecules. Since underivatized OPCs do not readily crystallize, a combination of analytical techniques may be used such as electronic circular dichroism spectroscopy [63], coupling constant analysis, DFT-based chemical shift calculations [64], DP4 analysis [65], and differential chemical shift (Δδ) measurements [64,66], to systematically narrow the stereoisomeric possibilities and complete the absolute configuration assignment. It should be emphasized that DFT calculations can support but not replace empirical observations.

In contrast to ^1^H and ^13^C NMR, HPLC-MS requires significantly less material and allows to analyze several substances during one separation, ensuring sample purity. Based on the mass-to-charge ratios and exact masses obtained from high-resolution mass spectra, HPLC-MS can determine the elemental compositions of compounds present in NEP extracts [67]. The most common source of ionization in such researches is electrospray ionization (ESI) [68]. Although ESI is highly effective for detecting high-molecular-weight species through the formation of multiply charged ions, its application to NEPs with a high degree of polymerization is limited, since it is difficult to interpret multiply charged ions [67,69].

To overcome this limitation, emerging techniques such as (matrix-assisted laser desorption ionization) MALDI coupled with time-of-flight (TOF) mass spectrometry have become increasingly important [57]. White et al. analyzed cranberry pomace dietary supplements using MALDI-TOF-MS and HPLC-ESI-MS. ESI-MS identified procyanidins from monomers up to hexamers (degree of polymerization = 6), while MALDI-TOF-MS detected oligomers up to degree of polymerization = 13, greatly improving structural assignment accuracy [70,71]. More recent work has demonstrated that, under optimized conditions, MALDI-TOF-MS can detect even higher oligomers, with reported values reaching degree of polymerization = 21 [72]; however, for most plant extracts, the practical detection range typically lies between degree of polymerization 12 and 15 [70,71].

**Figure 4:**
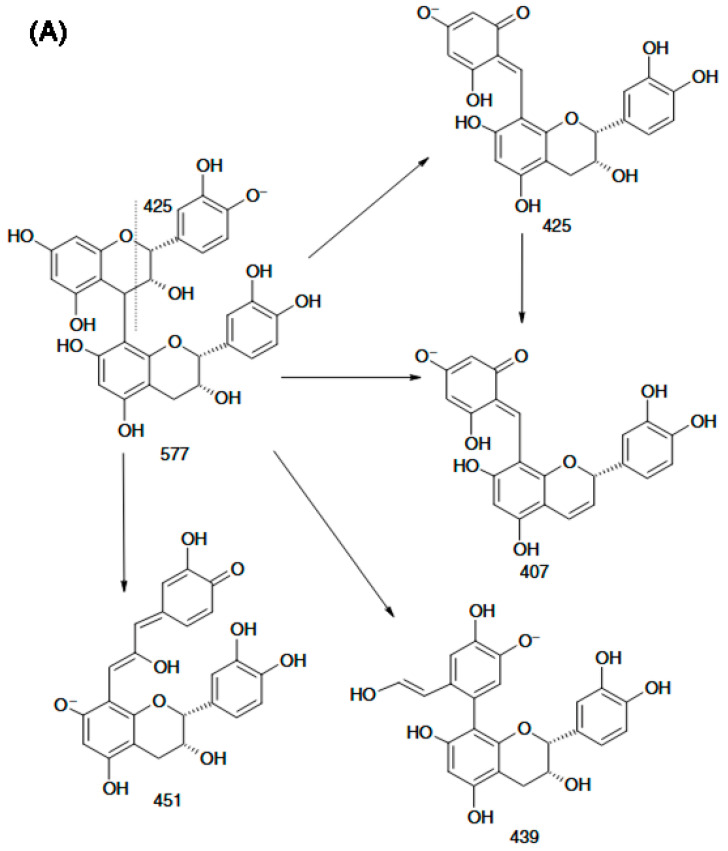

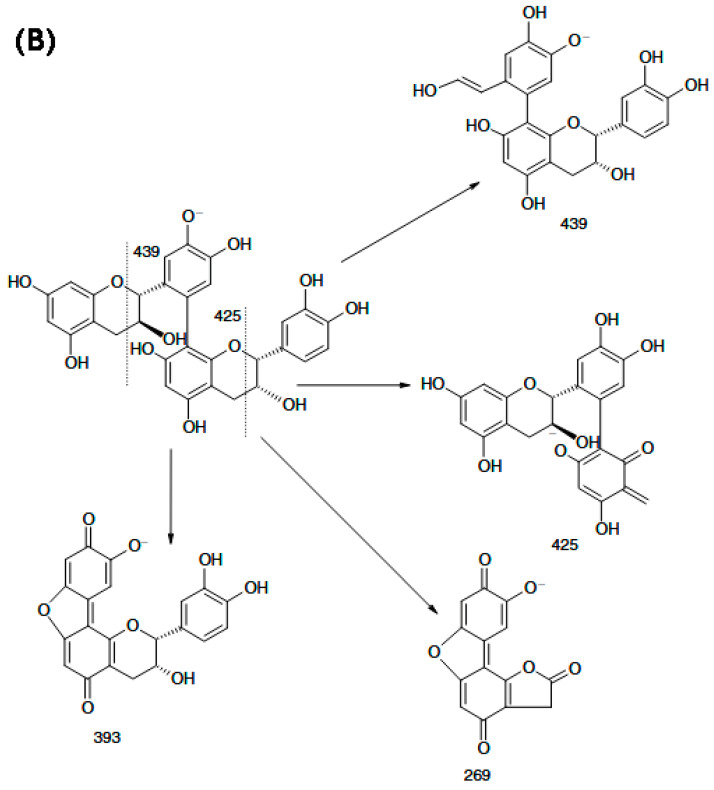
Scheme of the procyanidin B2 fragmentation in MS/MS experiment: (**A**) deprotonated procyanidin B2; (**B**) the fragment ions of a deprotonated dehydrodicatechin B_12_ with (−)-catechin and (+)-epicatechin units [73].

Compared with positive-ion ESI-MS, negative-ion mode provides higher sensitivity and more effectively suppresses interference from co-eluting species, while also generating highly diagnostic fragment ions from procyanidin dimers. These characteristic ions enable the development of a rapid approach to differentiate B-type procyanidins (*m*/*z* 451) from B-type dehydrodicatechins (*m*/*z* 393) in complex sample matrices. Figure 4A shows that the *m*/*z* 425 product ion readily converts to *m*/*z* 407 via loss of a neutral H_2_O molecule, while *m*/*z* 451 can produce *m*/*z* 289 through hydrogen abstraction. Moreover, unsubstituted phenolic hydroxyl groups on the aromatic rings are required to enable rearrangement through a quinone-type intermediate. Weixing Sun and Jack M. Miller reported the detailed structural features of dimers composed of (+)-catechin and (–)-epicatechin units linked through a C6–C8 interflavan bond, as well as the structures of their partial fragment ions. They further proposed that dissociation of the *m*/*z* 393 ion to yield the *m*/*z* 269 fragment likely results from 1,4-cleavage of the C-ring in the lower flavan-3-ol unit (Figure 4B).

**Figure 5:**
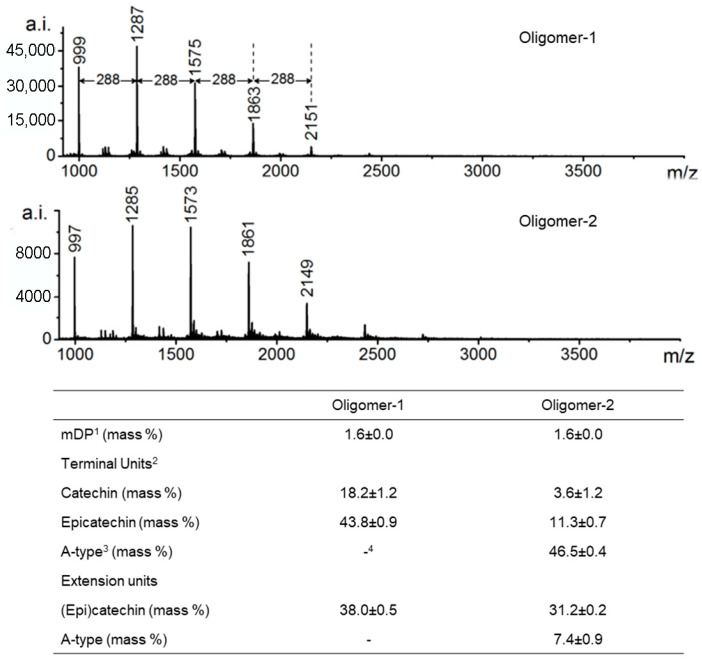
MALDI-TOF mass spectra of proanthocyanidins. Numbers on the peaks are the corresponding *m*/*z* of [M+Cs]^+^. ^1^ mDP: mean degree of polymerization; ^2^ Monomeric flavan-3-ols were included as terminal units; ^3^ A-type: subunits bonded by A-type interflavan linkages; ^4^: not detected. Adapted from [74].

HPLC-TOF-MS further enhances structural analysis of individual procyanidin molecules. Its high-resolution and accurate-mass capabilities allow clear differentiation between A-type and B-type linkages and identification of isomeric structures. The ability to determine exact molecular formulas is particularly valuable when distinguishing oligomers that differ by only 2 Da (e.g., 1287 Da vs. 1285 Da), such as those containing an additional interflavan ether bond characteristic of A-type procyanidins (Figure 5) [74]. Nevertheless, it should be noted that although MS methods are suitable for determining the structure of specific compounds [24,57], their utility for analysis of detailed conformational analysis is limited.

As mentioned above, the electronic circular dichroism data facilitate the direct assignment of the absolute configuration at the chiral center C4 and, thus, the interflavanoid linkage. The configuration of the interflavanoid bond at C4 can be assigned as R(4S) due to negative Cotton effects at 220–240 nm or as β (4R) in the case of positive Cotton effects at 220–240 nm [75,76].

### 2.1. A-Type Procyanidins

Unlike B and C-type procyanidins, which feature a single C-C interflavan linkage allowing rotational flexibility, A-type procyanidins A1 and A2 possess an additional ether (C-O-C) bond alongside the C-C bond, severely restricting conformational freedom and resulting in a single dominant conformation in the ground state. The A1 procyanidin structure was described by Kolodziej et al. [77] and recently confirmed by modern HPLC-MS, CD and low-temperature NMR data [78]. Characteristic NMR resonances at approximately δC = 115 ppm for C2′, C5′, and C6′ are consistent with a catechol ring bearing ortho-dihydroxylation in both the upper and terminal units. The ether bridge between C_2u_ and O_7t_ is identified by the chemical shift of C_2u_ at approximately δC = 100 ppm (δC = 98.9 ppm for A1 and δC = 98.8 ppm for A2), and by the less shielded resonance of C_7t_ at δC ≈ 150 ppm (150.8 ppm for both A1 and A2), reflecting the presence of the ether oxygen [78]. In NMR spectra, A1 and A2 differ primarily in the coupling constant of H_2t_. A1 exhibits ^3^J_HH_ = 8.86 Hz, corresponding to an anti-periplanar orientation of H_2t_ and H_3t_ (H_ax_–C_2t_–C_3t_–H_ax_), indicating a trans-configuration. In contrast, A2 shows ^3^J_HH_ = 1.35 Hz, consistent with a cis-configuration (H_ax_–C_2t_–C_3t_–H_eq_). CD spectra support these findings A1 displays a positive Cotton effect with [θ]_211_ = +45.732 and a shoulder at [θ]_238_ = +23.593, while A2 shows [θ]_211_ = +87.648 with a shoulder at [θ]_238_ = +48.783. Both compounds exhibit a positive Cotton effect for the 1La band between 200 and 220 nm, indicative of a β-orientation (4R stereochemistry) of the interflavan bond [63,78,79,80].

**Figure 6:**
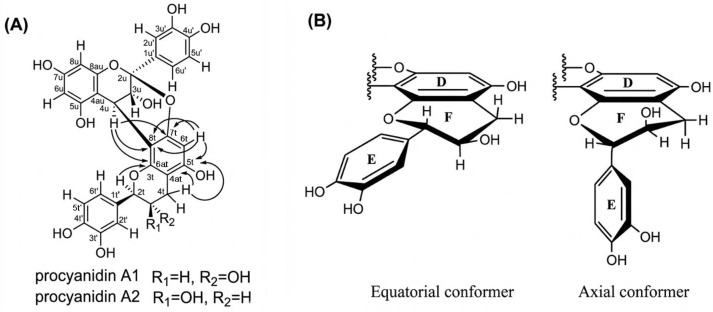
Chemical structures of procyanidin: (**A**) Important ^1^H-^13^C HMBC correlations indicated by solid arrows (→) of the A1 and A2 procyanidins [78]. (**B**) Equatorial and axial conformers of the lower part of the procyanidin A2 molecule [53].

In the case of A2 procyanidin, C and F heterocyclic rings are in half-chair conformation, while B and E rings lie in the equatorial plane with respect to the C and F rings, respectively (Figure 6B) [81]. This agrees with the data on theoretical bond lengths calculated using molecular mechanics using MM2 and MM3 and cluster analysis [59]. Theoretical calculations using DFT (M05-2X/6-31G** with SDM solvation method) indicate that the stability of the procyanidin A2 molecule is higher than the procyanidin A1 in aqueous and octanol solutions (free energy values ΔE_A2_ = 0 kcal/mol vs. ΔE_A1_ = 0.82 kcal/mol) [82].

### 2.2. B-Type Procyanidins

**Figure 7:**
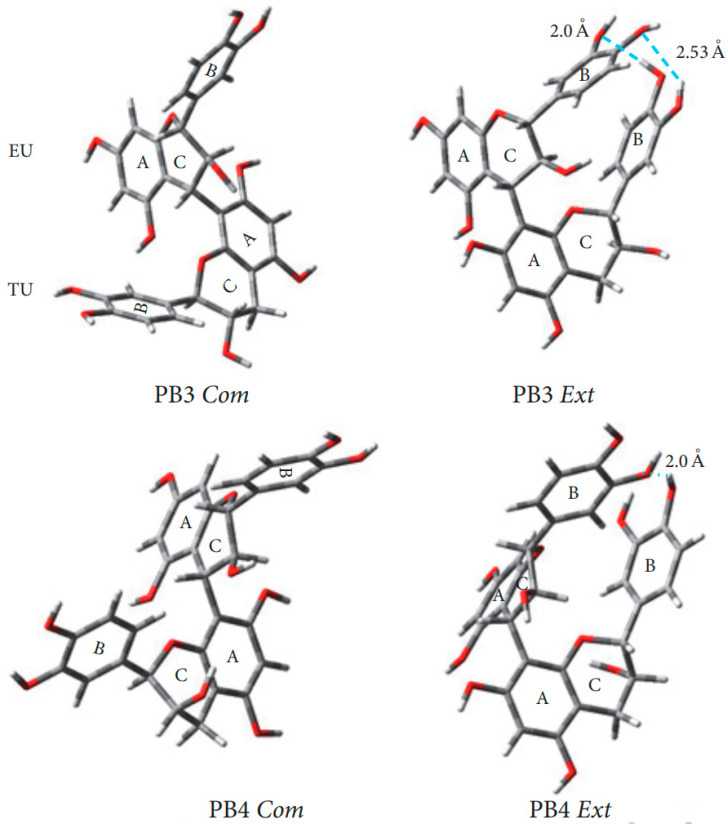
Optimized geometry of the procyanidin dimers in aqueous medium (SMD model) using the M05-2X/6-31G∗∗ DFT method. Com. compact conformation; Ext. extended conformation [85].

A comprehensive study of conformations B1-B4 procyanidins was published in 2006 [54]. In B-type procyanidins, the single C-C interflavan bond allows rotation, leading to distinct rotamers the compact rotamer, where the B rings are oriented oppositely, and the extended rotamer, where the B rings face each other (Figure 7) [83]. For procyanidins B1, B3 and B4 the predominant rotamer is compact, but the ratio of forms differs. Thus, procyanidins B1 [84] and B3 [84] are characterized by the dominance of a compact rotamer (95 5 in aqueous solutions), while for procyanidins B4 [53] the ratio is closer to the equivalent (76 24 in aqueous solution). For procyanidin B2 there is a slight bias in favor of the extended form (45 55 in aqueous solution).

**Figure 8:**
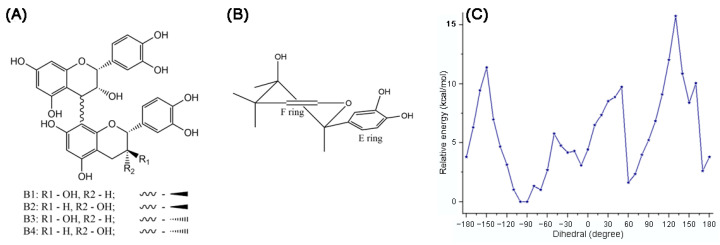
Planar structure of procyanidins B1–B4 (**A**) and preferred conformation of heterocyclic rings in procyanidin B2 (**B**); for ring C, the axial hydrogen in C4 position is substituted. Potential energy curve of B1 versus the dihedral angle θ (C3-C4- C8″-C8″a) in gas at the theoretical M06-2X/6-311G(d,p) level (**C**) [60].

Recent studies on B1 procyanidin correlate with earlier conformational analyses through calculations at the M06-2X/6-311G (d,p) level in gas phase, water, methanol, and benzene. Harmonic frequency analyses confirmed the stationary points and ZPE corrections, and solvent effects were modeled using polarizable continuum approach. The π-electrons were delocalized in the aromatic rings A1, A2, B1, and B2, but were absent in C1 and C2 (Figure 8). Loss of coplanarity between chromene and phenyl rings increased the dihedral angle θ (C3–C4–C8″–C8″a) to an average of −93.25°. Intramolecular hydrogen bonds were observed between 3′-OH and 4‴-OH, and between 3‴-OH and 5-OH, with the latter being more stable in weakly polar or nonpolar media. The global minimum conformer was found at −90°, while local minima at −70°, −10°, 60°, 150°, and 170° were 1.013–8.404 kcal/mol higher in energy (Figure 8C). Rotational barriers peaked at −150°, −80°, −50°, 50°, 130°, and 160°, with energies of 1.328–15.725 kcal/mol [60].

In addition to the experimental data obtained using NMR spectroscopy, calculations of stable conformations by the molecular mechanics method were carried out for all molecules, and in all cases the predicted and observed trends were similar the compact conformer dominates. The nomenclature describing the conformation of heterocyclic rings is as follows: Eq–Eq rings B and E are both in an equatorial position; Eq–Ax B in equatorial and E in axial position; Ax–Eq B in the axial position and E in the equatorial position; Ax–Ax: conformers were not found in the calculations (Table 1). Ax and Eq conformers are presented in Figure 6B.

The earlier reports are partially in conflict with the up-to-date data. Thus, the upper unit heterocyclic ring exists in an approximate half-chair conformation in both compact and extended rotamers [54]. However, coupling constants of the lower unit heterocycles reveal substantial axial orientation of the ring B. Line shape analysis of NMR spectra excludes equatorial and axial conformational interchange. These results and NOE experiments explain a skewed-boat conformation or between a half-chair and skewed-boat conformation for the terminal unit [53]. This conclusion is based on a discrepancy between the calculated constants of the equatorial and axial variants of the half-chair conformation for heterocycle and the NMR spectroscopy data. At the same time, in the case of B4 procyanidin, the heterocycle in the lower block is proposed to adopt a half-chair conformation. The extended rotamer is stabilized by hydrogen O-H ··C bonding between flavan subunits (the aliphatic hydroxy group at C-3_C_ with the aromatic hydroxy group at C-7_D_), while the compact rotamer can be stabilized by the intramolecular hydrogen bond between the aliphatic hydroxy group at C-3_C_ and the pyran oxygen of the ring F [53,60,85].

Procyanidin dimers exhibit distinct conformational preferences in aprotic organic solvents (e.g., acetone-d_6_ [53], dioxane-d_8_ [53], acetonitrile-d_3_ [86], DMSO-d_6_ [87]) versus water. According to NMR data and molecular modeling, such solvents favor elongated rotamers with greater interflavan torsional flexibility and reduced π–π stacking, yielding 70–80% elongated forms due to absent hydrophobic collapse, which stabilizes compact structures in water. The terminal C-ring adopts a relaxed half-chair or flattened conformation with low strain, while strong hydrogen bonding destabilizes compact structures, promoting axial-equatorial or equatorial–equatorial geometries. In water, conversely, disrupted intramolecular H-bonds enhance π-stacking in compact B-E ring rotamers [54,88].

Theoretical calculations of various conformational stabilities using molecular mechanics show qualitative agreement with experimental data in the case of procyanidins B1 and B4, while for procyanidin B2 this method is unreliable [54]. The report explains the above-mentioned phenomenon by the peculiarities of the calculation model, which does not always correctly take into account the influence of the solvent. As expected, subsequent DFT methods better capture solvent polarization and interflavan bond torsion barriers, addressing model-specific peculiarities [82,85]. B5-B8 procyanidins, due to the specificity of C4-C6 interflavan bond, have only one conformation in the ground state. In silico calculations reveal the pairwise structural similarity for B5 and B7 procyanidins and B4 and B8 procyanidins [85].

### 2.3. C-Type Procyanidins

Calculations of four possible conformations of C1 procyanidin ([(2*R*,3*R*,4*R*)-Flavan-3,3′,4′,5,7-pentol]-(4→8)-[(2*R*,3*R*,4*S*)-flavan-3,3′,4′,5,7-pentol]-(4→8)-[(2*R*,3*R*)-flavan-3,3′,4′,5,7-pentol]) using DFT (M05-2X/6-31G** with the SDM solvation method) were carried out by Mendoza-Wilson et al. Based on their computational analysis, the authors concluded that there are two stable rotamers with extended–compact and compact–compact conformations [82].

Recent studies show that there is no need to observe heteronuclear long-range interactions ^1^ H-^13^C in 2D HMBC spectra and record chemical shifts. It has been shown that ROESY spectra clearly reveal the configuration in C4 (α or β) and provide a spectroscopic method for distinguishing between C4→C8 and C4→C6 bound dimers or trimers of procyanidin without the need for derivatization [62,89]. The CD spectroscopy data confirm the β-configuration for both C-type procyanidins underscoring how such conformational equilibria, driven by steric constraints and intramolecular hydrogen bonding [62].

C2 procyanidin exists in four conformations in aqueous solution, one of which dominates (~60%) [83]. The heterocyclic rings C and F (upper and middle blocks) for the dominant component adopt an equatorial conformation, while the heterocyclic ring I (lower block) adopts a predominantly axial conformation, which allows the formation of an α-interflavan bond. This isomer is Eq/Eq/Ax, which corresponds to the previously obtained data for peracetylated C2 [90].

Analyses of the calculated surface areas indicate that conformers with smaller exposed surface areas are more stable (Table 2) [82,85]. In such configurations, the molecule becomes less accessible to interactions, most of the hydroxyl groups are oriented inward. It can be assumed that the reactivity of B2 and B4 procyanidins will be higher than that of B1 and B3. Since this pair is characterized by a small difference in the energies of the compact and extended conformations, a smaller amount of energy is required to transfer the molecule to a reaction-accessible conformation.

According to the ^1^H NMR spectral data, a high energy barrier regulates rotation around the interflavan bond in B3 and B4 procyanidins (two separate isomers can be observed), whereas a relatively low barrier is observed in B1 and B2 procyanidins (peak broadening prevents the resolution of two separate isomers). This behavior appears to be linked to structural differences in B1 and B2, the upper unit is epicatechin, while in B3 and B4, it is catechin. Previous studies on related systems have shown that such effects are associated with the presence or absence of steric hindrance during rotation around the interflavan bond, determined by the 2,3-cis or 2,3-trans configuration of substituents, respectively. Interestingly, these rules established for dimers do not apply to trimers. In the case of procyanidin C2, composed entirely of catechin units, large differences were observed in the rate of rotation. These findings indicate that, for trimers, conformational exchange is primarily governed by ring deformation rather than interflavan rotation [91,92].

The number of possible conformations of procyanidins increases sharply when moving from dimers and trimers to more complex oligomers, which greatly complicates the search for the global minimum. For such systems, an effective strategy involves generating a set of initial rotamers, performing preliminary optimization at the molecular mechanics level, and then carrying out final optimization using DFT methods. This approach captures a broader range of relevant low-energy minima and reduces the likelihood of missing stable conformers in calculations of tetramers and larger structures.

For example, molecules of epicatechin pentamer (Epi-5), arecatannin A3 (ATA3), and catechin pentamer (Cat-5) can each adopt up to 16 distinct conformations. These initial geometries were optimized without restraints using DFT at the B3LYP/6-31(d) level in the gas phase. Notably, the lowest-energy conformers of Epi-5 and ATA3 exhibit the same rotamer pattern, Co-Co-Ex-Ex, along the C-4:C-8 bonds, whereas the Cat-5 lowest-energy structure is characterized by the Ex-Ex-Co-Ex arrangement. Moreover, the three-dimensional distribution of hydroxy groups in the lowest-energy conformers of Epi-5 and ATA3 is similar but differs from that of EGCG. This suggests that Epi-5 and ATA3 may interact with target biomolecules through position-specific hydrogen bonding, thereby contributing to their anticancer activity via mechanisms distinct from EGCG, such as the suppression of PC-3 prostate cancer cell proliferation and invasion. In contrast, Cat-5 does not exhibit this activity [93].

### 2.4. Conformations and Properties Relationships

Despite the detailed study of procyanidin structures, relatively few works address the relationship between their structural features and functional properties. In most cases, the authors consider correlations between different classes of flavonoids, paying almost no attention to the differences between members of the same group. For example, in a comprehensive evaluation on the antioxidant properties of various flavanoids depending on their structure, it was proposed that for high biological activity was shown that a catechol moiety on ring B is required for decent activity, as the –OH group in C3 position can function as a chelation site. It was established that the presence of a C2-C3 double bond near the 3-OH group increases the chelating activity [94]. Therefore, procyanidins containing a phenolic group at C3, though not optimal, can act as effective inhibitors of oxidative processes, which has been confirmed in numerous studies [33,95,96,97,98,99,100,101,102,103,104]. Despite these works do not address the differences in the properties of individual procyanidins, several trends across antioxidant, neuroprotective, antibacterial, and enzyme-inhibitory contexts can be defined. Three structural factors consistently emerge as the primary determinants of biological activity: interflavan linkage type (C4-C8 vs. C4-C6 and orientation α/β); molecular conformation (compact vs. extended); and degree of polymerization, with a secondary contribution from galloylation.

The DFT results showing that C4→C8 linked dimers (e.g., B3 and B4) in their Compact conformations exhibit the strongest electron-transfer and H-atom donation ability agree with experimental findings that C4-C8 bonded dimers are generally more efficient radical traps than dimers with C4→C6 interflavan bonds [85]. Dimers B1-B4 adopt more closed conformations and display slightly higher hydrophobicity, promoting stronger interactions with lipid-derived radicals and enhancing LDL-protective antioxidant activity [104]. Among all B-type procyanidins B8, characterized by a C4α→C6 linkage and Extended conformation, showed the lowest activity [85].

Studies in cell and zebrafish models reported stronger biological protection with increasing degree of polymerization, with trimer C1 outperforming dimers and monomers in suppressing ROS, MDA, and restoring antioxidant enzyme activity [105,106,107]. The DFT results reveal structural trends that mirror these biological outcomes: dimers with more favorable electron-donating configurations (e.g., B3 and B4) would be expected to outperform monomers but be surpassed by trimers, which offer a greater density of hydroxyl groups and more extensive delocalization networks [105,106,107].

Galloylation has been repeatedly shown to enhance activity. Reported superior neuroprotective effects for galloylated dimers (B1-G, B2-G) described a dramatic increase in radical-trapping efficiency for the galloylated dimer B2-3-O-gallate compared to its non-galloylated counterparts [104,105,106,107]. Although the present DFT work did not model galloylated species, the electronic rationales are congruent: galloyl groups extend the π-system and stabilize phenoxyl radicals, consistent with the polarization and frontier orbital effects captured computationally in [85].

The antibacterial and enzyme-inhibitory properties of procyanidin dimers reported in broader literature often depend on the same structural determinants. More compact B1-B4 isomers usually show stronger interactions with bacterial membranes or enzyme active sites due to greater hydrophobic surface complementarity, whereas B2-B8 dimers, being more elongated and polar, often display lower potency. The conformational dependence observed in Fukui indices, where C4β→C6 B5 (Compact) was most susceptible to electrophilic attack while C4α→C6 B8 (extended) was the least [85]. Likewise, enzyme inhibition studies frequently show that galloylated or more compact dimers exhibit stronger inhibitory constants, which the DFT-derived HOMO localization and charge distribution patterns help rationalize.

In recent years, there has been a lot of research on interactions between procyanidins and different proteins that significantly influence their bioactivity and bioavailability. These interactions can be broadly categorized into non-covalent and covalent mechanisms, each driven by distinct chemical forces and environmental conditions. Non-covalent interactions, primarily mediated by hydrogen bonding [108], hydrophobic effects [109], and van der Waals forces, allow reversible binding at low to neutral pH that often leads to protein aggregation or conformational changes without forming new chemical bonds [110]. In contrast, covalent interactions involve irreversible adduct formation, typically through oxidation of procyanidins to quinones or semiquinones, which react with nucleophilic sites on proteins such as lysine, cysteine, or tryptophan residues via Michael addition or similar pathways [111,112]. Recently, Zhang et al. specifically focused on covalent interactions, highlighting their mechanisms, structural consequences, and functional implication [113]. This distinction is crucial, as non-covalent binding preserves the native structures to a greater extent, facilitating dynamic equilibria in biological environments, whereas covalent adducts can permanently alter protein functionality, enhancing stability but potentially reducing enzymatic activity or allergenicity.

**Figure 9:**
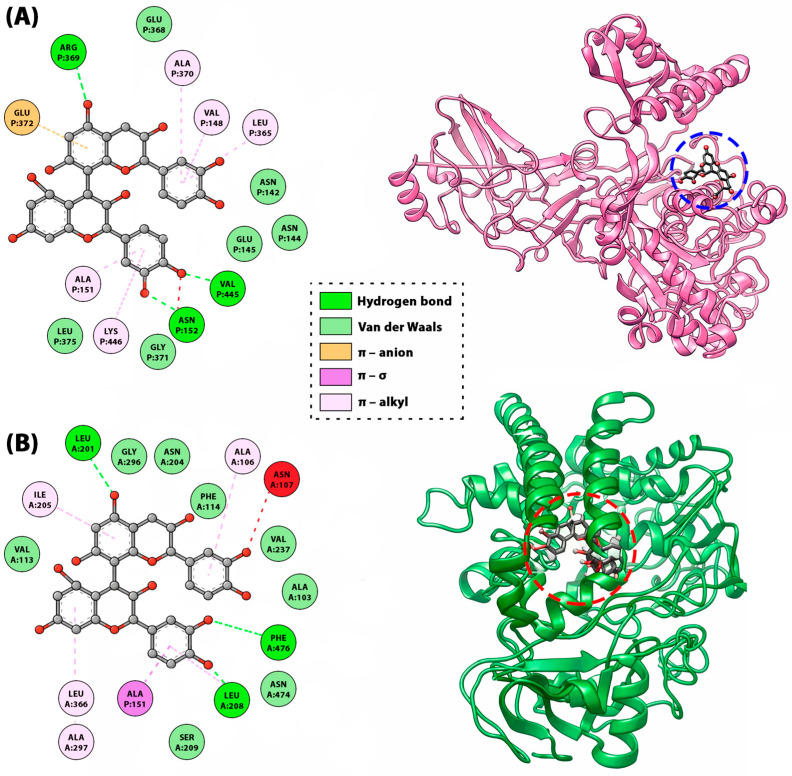
The 2D map of amino acid-ligand interactions and the positions of the procyanidin B1 on the urease (**A**) and cytochrome P450 (**B**) enzymes suggested by molecular docking studies [114].

A prominent example of non-covalent interaction is the binding of procyanidin B1 to human tyrosinase enzyme (Figure 9). Binding orientation analysis indicated an array of hydrogen bonds as contributed by several aminoacids. It initiated van der Waals and π–π stacked interaction, respectively (Figure 9). Remarkably, OH groups of two phenyl rings directly participated forming hydrogen bonds with the essential residues which contributed to the malfunction of the targeted enzyme. These results (binding energy −12.84 kcal/mol) imply that B1 might effectively inhibit the human tyrosinase enzyme [60]. Another example of non-covalent interactions is the binding of procyanidin B2 (a B-type dimer) to urease and cytochrome P450 enzymes. For urease (Figure 9A), procyanidin B1 shows a binding energy of −11.1 kcal/mol versus −3.2 kcal/mol for thiourea, forming three hydrogen bonds with distances of 2.17–3.10 Å together with multiple π–alkyl and one π–anion interaction, consistent with an extended, polar binding mode. In cytochrome P450 (Figure 9B), the same ligand exhibits a stronger score of −12.8 kcal/mol, far exceeding the reference antioxidants ascorbic acid (−5.5 kcal/mol) and BHA (−6 kcal/mol), with interactions dominated by hydrophobic π-contacts, indicating a deeply buried, compact conformation in the catalytic pocket [114]. The interaction occurs at specific sites on BSA, involving tryptophan residues (Trp-134 and Trp-212), leading to moderate affinity that is higher than monomeric epicatechin but lower than larger oligomers like cinnamtannin A2. Similar non-covalent aggregation is observed with salivary proteins, where procyanidins like B-type dimers contribute to astringency by precipitating proline-rich proteins through multivalent hydrophobic and H-bonding interactions, influencing sensory properties in foods like wine or chocolate [9].

## 3. Synthesis and Extraction of Procyanidins

There are two fundamentally different approaches to the preparation of procyanidins direct synthesis of target substances and isolation from natural sources. Depending on the problem to be solved, one or another method may be used. Extracts derived from natural materials are complex mixtures of numerous bioactive components and can be used as dietary supplements or pharmaceuticals without extensive purification. However, for fundamental studies on bioavailability and specific physicochemical properties, the isolation of individual compounds is essential. Current highly selective purification methods yield only milligram quantities of target compounds, while fundamental research often requires hundreds or even thousands of milligrams. To address this limitation, direct synthetic methods for individual procyanidins are being actively developed [115,116,117,118,119,120]. A number of reviews [95,115,116,118,119,120,121] are devoted to the preparation of procyanidins, nevertheless, the synthesis of only one type of procyanidin is described in detail. This review aims to provide a comprehensive overview of the available strategies for their preparation.

### 3.1. Synthetic Methods

The major synthetic strategies for various procyanidins are summarized in Table 3. Frequently used synthetic strategies rely on phenol protection, activation with a Lewis acid (or equivalent), and the introduction of a halogen at the C8 position to prevent the formation of self-condensation products and multiple undesirable reactions [122]. Trimethylsilyl trifluoromethanesulfonate, AgBF_4_, TiCl_4_, SnCl_4_, and BF_3_ Et_2_O, etc., are used as Lewis acids in the condensation of catechin subunits [123,124]. Benzyl protection is widely used to protect phenolic groups. It can be removed at the last stage by a hydrogenolysis reaction, which usually proceeds cleanly and efficiently. Other widely used protecting groups include methoxymethyl, benzyloxymethyl, tosyl, and benzenesulfonyl, but not all of them can be removed from target products in a similar way [125]. However, since there are a significant number of reviews devoted to the synthesis of both OPCs and polymeric procyanidins (PPCs) [28,115,126,127,128], we see no necessity to discuss this in detail.

### 3.2. Isolation from Natural Sources

Nowadays, the main industrial sources of procyanidins are grape seeds (*Vitis vinifera*) and pine bark (*Pinus radiata*, *Pinus maritima*), due to its favorable economic indicators [18,31,141,142]. Although procyanidins are present in many other plants, their content typically does not exceed 0.1–0.5% of the raw material weight, which contributes to the high cost of the final product. This creates a strong incentive to identify new, accessible, and inexpensive sources of raw materials, as well as to develop efficient production technologies.

Among promising crops for procyanidin extraction are apples [11] (*Malus domestica*), cocoa beans [13] (*Theobroma cacao*), common beans [12] (*Phaseolus vulgaris*), grapes [143] (*Vitis vinifera*), peanuts [144] (*Arachis hypogaea*), plums [145] (*Prunus domestica*) and hop [146] (*Humulus lupulus* L.) (Table 4). The procyanidin content in these materials ranges from 1.5 to 35.3 mEq catechin/g dry matter. It can vary depending on plant variety, age, and tissue type. For example, grape berries contain only 0.05 mEq/g, whereas grape seeds reach 35.3 mEq/g, similarly, soybean seed coats contain much higher levels than the seeds themselves [147]. Besides cultivated plants, procyanidins can be isolated from agricultural wastes [15], which are generated rapidly with an average annual increase rate of 5–10% [148]. Among the non-edible parts of the plants are roots, bark, peel, pomace, husks, bagasse, etc. Lignocellulose residue after extraction of procyanidins can be further utilized as an appropriate precursor to produce the low cost activated carbons [149,150,151,152].

**Figure 10:**
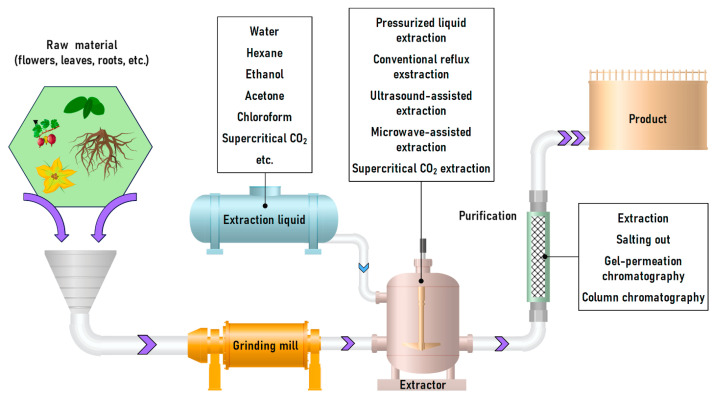
Principal scheme for procyanidins obtaining from raw plant materials shredding to extraction and extract depuration/purification.

The general scheme for large-scale procyanidin extraction from plant raw materials involves grinding the material followed by extraction (Figure 10). The resulting bioactive extract is purified from toxic and harmful substances. It should be noted that when extracts are used as dietary supplements, the isolation of individual compounds is typically not performed [157].

Extraction of proanthocyanidins from wood raw materials is usually carried out by repeated sequential extraction with water at temperatures of 70–90 °C. To improve the recovery of oligomeric tannins and obtain low-viscosity extracts, salts such as NaS_2_, Na_2_S_2_O_5_, and Na_2_CO_3_ can be added [158]. The resulting extract is then concentrated to 30–40% solids content and either spray-dried or vacuum-dried to 80–90%, after which it is packaged at 80 °C. Upon cooling, the extract solidifies. Details of the industrial process are provided in the work of Pizzi et al. [159] (Figure 10).

Laboratory research in the field of procyanidins obtaining from natural raw materials is mostly focused on optimizing extraction processes [160]. Conventional approaches are based on the use of organic solvents such as absolute methanol [161], absolute ethyl acetate [162], 70–80% ethanol solution [14,163,164], solution of acetone and water in various proportions [165,166,167]. The extraction of procyanidins occurs at temperatures from 0 to 100 °C, while the preferred range is from 10 to 55 °C [4,18,168]. The solubility of procyanidins is determined by the polarity of the solvent for example, in the least polar ethyl acetate only OPCs can be dissolved. Since procyanidins are stable at pH 2–6, in some experiments, acidified aqueous–organic mixtures were used to increase the extraction rate [143,169].

The disadvantages of traditional methods include long extraction time, high solvent consumption, low selectivity and restrictions on the use of certain solvents in the food industry. In this regard, a number of researchers have recently resorted to combined extraction methods of procyanidins ultrasonic-assisted extraction (UAE) [7,170,171,172,173,174], (Table 5) microwave-assisted extraction (MAE) [175,176,177,178,179], pressurized liquid extraction (PLE) [180,181,182], extraction with supercritical fluids [11,183,184,185,186] and extraction with deep eutectic solvents (DES) [187,188,189].

#### 3.2.1. Ultrasonic-Assisted Extraction

**Figure 11:**
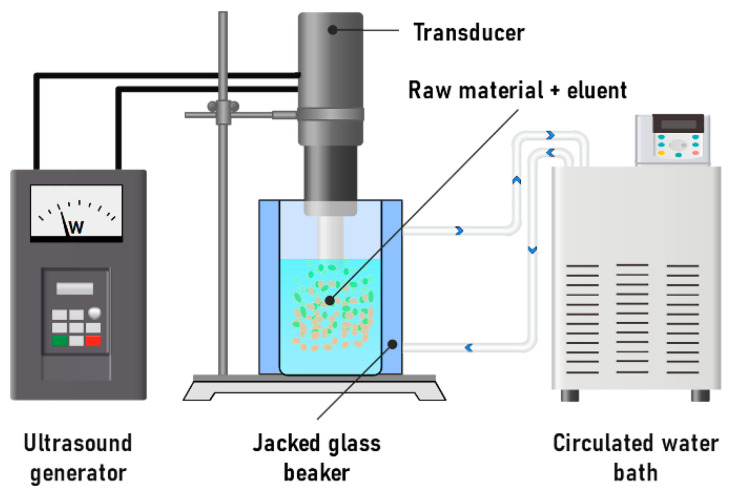
Scheme of a laboratory setup for the ultrasonic-assisted extraction of procyanidins.

It has been shown that the traditional extraction method requires significant solvent consumption [191]. However, the use of ultrasonic-assisted extraction can significantly reduce extraction time and decrease the need for water–ethanol solution (Figure 11). This method relies on the disruption of cell walls through ultrasonic vibration. Ultrasonic waves disturb the boundary diffusion layer, enhancing the penetration of the solvent into the plant matrix. As a result, the raw material swells more rapidly, and the formation of turbulent and vortex flows improves mass transfer, accelerating the overall extraction process [192,193]. This method is characterized by a short process time and minimal environmental impact, which is used in the processing of food industry waste to obtain various polyphenols, including procyanidins [171].

In UAE processes, an increase in the yield of oligomeric and monomeric fractions of procyanidins is observed. Thus, the investigation of procyanidins disaggregation under the action of ultrasound with frequencies of 20 to 45 kHz indicates that the use of this method can increase the extractability of flavanols, OPCs and PPCs by 49, 41 and 35%, respectively, compared with extraction without ultrasound assistance [194]. At the same time, the use of high frequencies of about 40–45 kHz leads to a decrease in the concentration of the monomeric fraction compared to the experiment at 20 kHz. The authors explain this phenomenon by the formation of a large number of microbubbles during irradiation, which prevented mass transfer. The observed increase in antioxidant activity appears to be related to the breaking of bonds between procyanidins and polysaccharides [194]. In most cases, an ethanol concentration of 60–70%, a temperature of 50–70 °C, and an extraction time of approximately one hour are used [194,195].

There are a number of modifications of the UAE method. One of them is applying of high-intensity, low-frequency ultrasound, which further reduces the extraction time [172]. Another modification is the combination of UAE with macroporous resin, which not only reduces the process time, but also increases the extraction yields [173].

#### 3.2.2. Microwave-Assisted Extraction

**Figure 12:**
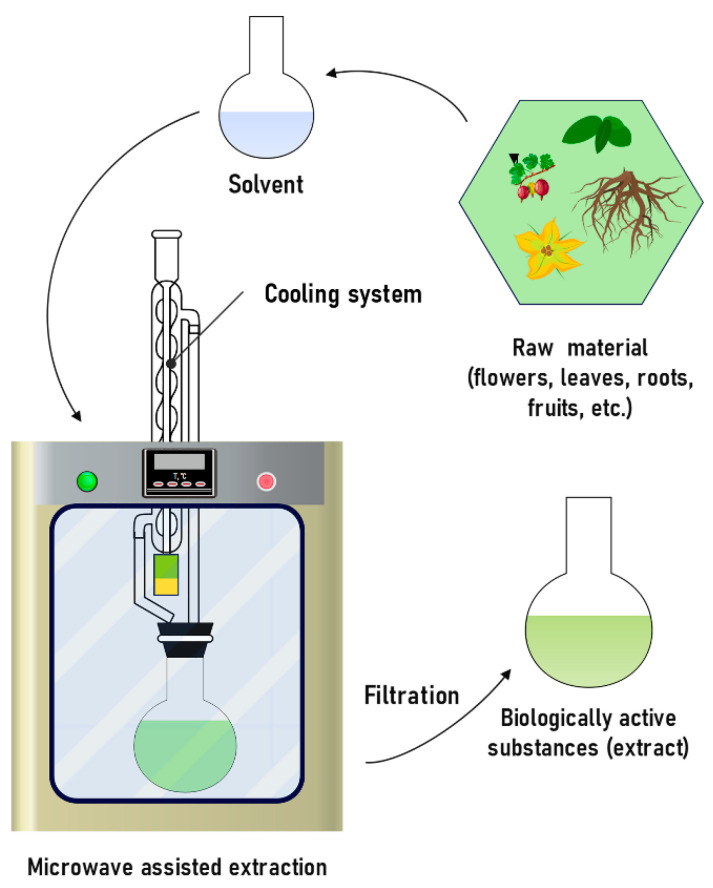
Schematic diagram of microwave-assisted extraction process.

MAE is widely used to extract polyphenols, proanthocyanidins, and flavonoids [175,176,177] (Figure 12). The mechanism is to transfer heat to the solvent using frequencies in the range from 300 MHz to 300 GHz, which contribute to the destruction of the cellular structure. The interaction between solvent molecules and released compounds is enhanced due to the formation of pores in the solid, which promotes fast mass transfer and leads to an increase in the efficiency of the extraction process [196]. The advantages of this method are a high percentage of extraction of the substance, minimal use of solvent, and a 10–20-fold reduction in the extraction time [178,179,197]. Researchers have identified various factors that affect the yield of substances using this method, which include temperature, time, solvent type, ratio (solvent/solid) and power [198,199].

The disadvantages of the method include the high cost of the equipment used and the near-complete inability of extracting heat-sensitive components due to the rapid and uncontrolled local heating of sample parts. The second drawback can be mitigated by using microwaves at the sample pretreatment stage, rather than at the extraction stage [200]. To do this, the sample is placed in a microwave device without adding an organic solvent for 1–2 min, and then an organic solvent is added to the sample for further extraction. Despite the simplicity of the described procedure, no experimental studies have been reported of its application.

#### 3.2.3. Carbon Dioxide Supercritical Extraction

**Figure 13:**
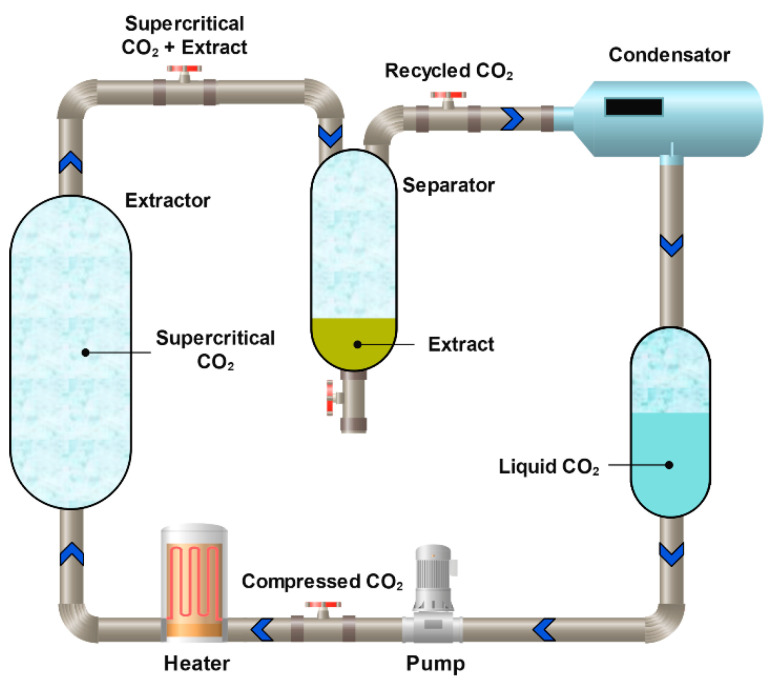
Schematic diagram of the process of extraction with supercritical CO_2_.

Extraction with supercritical CO_2_ is a supercritical fluid extraction method in which liquefied CO_2_ is used as a unique solvent [201] (Figure 13). This approach is widely applied both to the extraction of various flavonoids [202] and to the isolation of procyanidins [184,185], including those obtained from agricultural waste. Because supercritical CO_2_ is a non-polar solvent, polar co-solvents such as water, ethanol, or methanol must be added during procyanidin extraction [203,204]. The process can be performed in three stages, e.g., pure CO_2_, a MeOH–CO_2_ mixture, and pure methanol. In the first stage, the oil (non-polar) fraction is separated, while the second and third stages allow the isolation of target polyphenols, with an overall yield of approximately 50% [205].

Supercritical CO_2_ extraction is considered one of the most promising methods for the large-scale production of procyanidins from natural sources. Da Porto et al. demonstrated that the use of supercritical CO_2_ was several times more efficient than in a pilot-scale industrial setup total procyanidin recovery reached 8.1 mEq/g with supercritical CO_2_ extraction compared to 0.7 mEq/g with UAE [184]. Despite its excellent efficiency in recovering OPCs, this method remains the most expensive among current extraction technologies. Closely related, more cost-effective alternatives include pressurized liquid extraction and subcritical water extraction [206,207,208]. At present, pressurized liquid extraction is the most economically viable of these methods [209].

Phenolic compounds were extracted from grape marc using supercritical fluid extraction CO_2_ with an ethanol/water co-solvent, and 8 MPa was identified as the optimal pressure. At this pressure, varying co-solvent concentrations and CO_2_ flow rates were tested, with the best yields of proanthocyanidins achieved at 4 kg/h CO_2_ related to 7.5% EtW and 6 kg/h CO_2_ for 10% EtW. The 6 kg/h for 10% EtW setup yielded higher levels of monomeric and oligomeric proanthocyanidins and greater antioxidant activity. Results were compared to conventional methanol extraction [53].

Grape seed residues left after supercritical CO_2_ extraction contain about 3.8 g/100 g of procyanidins and can serve as a valuable source. A high-purity extract (over 94% procyanidins) can be obtained using AB-8 resin. Microencapsulation using Arabic gum and maltodextrin (40 60 ratio) with a 30 70 core-to-wall ratio and 20% slurry content yields a productivity of 89.7% and encapsulation efficiency of 99.2% [210].

Supercritical CO_2_ extraction has been used to isolate proanthocyanidins from grape seeds, with pressure, temperature, and ethanol concentration as key variables. Ethanol content had the greatest influence on extraction efficiency. Due to differences in polarity, each compound reached its maximum yield under specific conditions. For example, gallic acid, epigallocatechin, and epigallocatechin gallate were best extracted at 300 bar, 50 °C, and 20% ethanol [211].

High yields of procyanidins and proanthocyanidins were obtained from *Arachis hypogaea* (peanut) skins using supercritical CO_2_ with ethanol as a co-solvent. Solubility models were employed to describe compound behavior under various conditions, and response surface methodology was used to optimize pressure, temperature, and ethanol flow rate. Optimal conditions of 20.4 MPa, 333 K, and 0.17 mL/min resulted in 2.32 mg/g of procyanidins and 0.4 mg/g of proanthocyanidins [212]. Babova et al. investigated the extraction of antioxidant-rich anthocyanins and phenolic compounds from bilberry (*Vaccinium myrtillus*) using a two-step method supercritical CO_2_ extraction followed by subcritical CO_2_ extraction, both enhanced with 10% ethanol as a co-solvent. This strategy improved both efficiency and selectivity of bioactive compound recovery [213]. Blanch et al. studied the effect of CO_2_ treatment on the flavonoid profiles of strawberries (*Fragaria vesca* L.) using Q-TOF and HPLC quadrupole analysis. CO_2_-treated fruit, particularly at 20% CO_2_, exhibited increased levels of proanthocyanidins B1 and B3 and higher flavonol content. Unlike air-treated strawberries, anthocyanin levels remained stable. The induction of catechins under CO_2_ likely contributes to reduced fungal decay [214]. Dry ice treatment promoted the extraction of high-molecular-weight skin proanthocyanidins. Low-temperature prefermentative maceration increased overall proanthocyanidin levels but unexpectedly favored seed-derived compounds. Enzymatic maceration consistently enhanced proanthocyanidin concentration throughout fermentation by boosting both skin and seed phenolic extraction [215]. Hayrapetyan et al. explored supercritical CO_2_ technology for sustainable grape pomace valorization with minimal environmental impact. Supercritical CO_2_ with water as a co-solvent effectively extracted phenolics and fibers, including pectins. The molecular weights of the extracts were approximately 478 ± 6 kDa (SC-60) and 449 ± 3 kDa (SC-80 + H_2_O), demonstrating the potential of supercritical CO_2_ as an eco-friendly waste utilization method [216].

#### 3.2.4. Pressurized Liquid Extraction

PLE offers several advantages over conventional extraction methods, including short dwell times and reduced solvent consumption. Compared to supercritical extraction, it is characterized by lower equipment costs. The process is carried out in a closed, inert system under heating, which ensures rapid mass transfer and enhances the dissolution of plant material in the solvent medium [217]. It is important to note that subcritical solvents (for example, water–alcohol mixtures) are less efficient than conventional solvents for extracting high-molecular-weight procyanidins with a degree of polymerization n > 5. However, they are significantly more effective for low-molecular-weight procyanidins (n = 2–5) [218].

**Figure 14:**
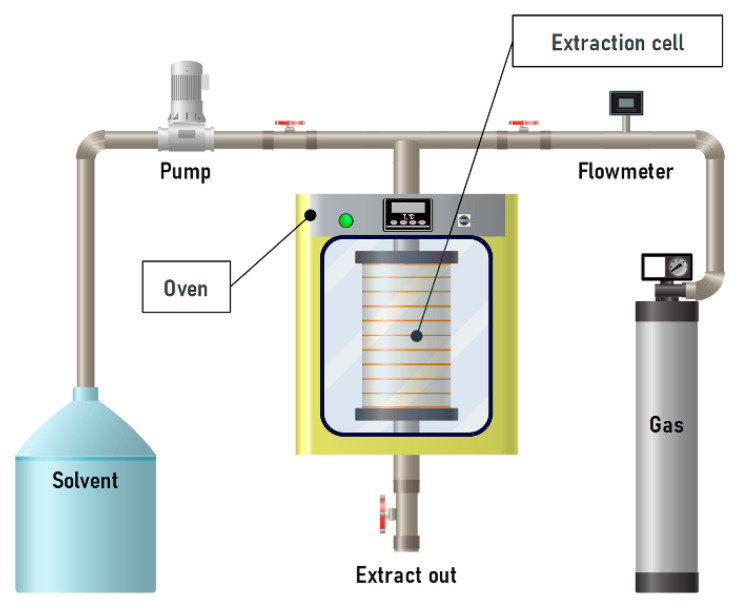
Schematic diagram of the installation for liquid extraction under pressure.

OPCs can be extracted by enzymatic methods, which, when combined with PLE, significantly increase extractability and allow the recovery of non-extractable polyphenols [28,219]. The efficiency of the process depends on temperature, surface tension and viscosity of the solvent, and extraction time. For example, Okiyama et al. performed extraction of cocoa bean shells at 60, 75, and 90 °C for 5, 30, or 50 min under 10.3 MPa (Figure 14). The highest yield of procyanidin B2 (1.94 mEq/g) was achieved at 90 °C with a 30 min extraction time. With longer exposure, the yield decreased to 1.46 mEq/g, which the authors attributed to thermal degradation of the compound [181].

The main advantages and disadvantages of the described extraction methods are summarized (Table 6). UAE can be recommended as a simple, inexpensive, and relatively effective technique for procyanidin extraction. Although MAE is highly efficient and easy to apply in laboratory settings, it is unsuitable for extracting heat-sensitive components and difficult to scale up for industrial applications. Supercritical CO_2_ extraction provides the highest efficiency but requires very expensive equipment. PLE represents a more cost-effective alternative with comparable efficiency, making it more practical for large-scale applications.

#### 3.2.5. Extraction with Deep Eutectic Solvents

The use of DES represents one of the most promising modifications of the extraction methods described above. These solvents consist of a hydrogen bond donor and an acceptor [220] and are widely applied for extracting natural compounds, including phenolic compounds [221]. When combined with MAE, UAE, or PLE [188,189,221,222], DES generally enhances extraction efficiency, as confirmed by the majority of studies. For example, Loarce et al. reported that combining a choline chloride oxalic acid (1:1) DES with pressurized hot water extraction (PHWE) increased total anthocyanin yield from grape pomace by a factor of four compared to conventional PHWE [188]. Similarly, Neto et al. described a DES–MAE technique for proanthocyanidin extraction from grape pomace, which not only increased the extraction yield by 238% compared to conventional ethanol extraction but raised the average degree of polymerization from 6 to 7.37 [223].

However, some studies have shown that conventional solvents may outperform DES in certain cases. Zannou and Koca found that using a choline chloride urea DES for the extraction of blackberry anthocyanins resulted in no detectable anthocyanins in the final extract [187]. Panic et al. reported no significant difference in the extraction yields of anthocyanins from grape pomace when comparing choline chloride-based DES with acidified aqueous ethanol [224].

Overall, the application of DES remains a promising but controversial approach that requires further investigation and optimization for specific targets and matrices. The latest developments in this field are summarized in a recent review by Foroutani et al. [189].

#### 3.2.6. Non-Extractable Procyanidins

Non-extractable procyanidins are a particular case of non-extractable polyphenols (NEPs) which is composed of molecules with diverse molecular weights, such as phenolic acids (low), hydrolyzable tannins (intermediate and high), and proanthocyanidins (intermediate and high). NEPs interact with the food matrix, especially polysaccharides and proteins, through various mechanisms: hydrogen bonding (between the hydroxyl groups of phenols and the oxygen atoms of the ether crosslinks), hydrophobic interactions and covalent bonds [225]. Hydrogen bonds are found between the food matrix and non-extractable proanthocyanidins and hydrolyzable tannins [225]. Covalent bonds are present bonding phenolic acids [226] and possibly non-extractable proanthocyanidins and hydrolyzable tannins. Thus, these compounds are mostly highly polymerized polymers or mono- or oligomers bound to polysaccharides or proteins [227], which, due to their large molecular weight and complex structure, are practically not recoverable by common extraction methods [228].

Recent quantitative investigations have shown that non-extractable polyphenolic compounds (NEPCs) often exceed the extractable fraction in many fruits and cereals. As summarized in Table 7, apples [178,229], grapes [230], and related matrices can contain up to 288 mg/100 g DW of non-extractable polyphenols, including proanthocyanidins [178], representing as much as 70% of the total phenolic content [69]. A more detailed compendium of NEP extraction yields across different plant materials has been published elsewhere [68]. However, NEPs isolation involves multiple steps that are not standardized and strongly depend on the physicochemical characteristics of the plant material [67,68,231,232]. Although a range of analytical techniques has been employed for NEP characterization, their specificity remains limited, and no universally accepted quantification protocol has yet been established [231]. Efficient NEP recovery requires disruption of their interactions with the plant matrix [233]. This is currently achieved by chemical or enzymatic depolymerization, most commonly through acid, alkaline, or enzyme-assisted hydrolysis [234]. Acid–base treatments are simple and rapid, yet certain phenolic structures show low stability under extreme pH conditions, which may lead to partial degradation of target compounds.

Acid hydrolysis to break the glycosidic bond can be carried out in two ways hydrolysis with methanol/H_2_SO_4_ to extract NEPs–hydrolysable polyphenols [236] or hydrolysis with butanol/HCl/FeCl_3_ to determine the content of non-extractable proanthocyanidins [237]. It is worth noting that there is no consensus on the efficacy of method. Despite the long period of application of both methods, there are very few studies comparing them and their results are contradictory [238,239]. The alkaline pH can induce cleavage of the ether or ester bonds between phenolic acid and the cell wall, which further increases the release of phenolic compounds from polysaccharide structures [240]. The most commonly used bases for alkaline treatment are sodium hydroxide, ammonium hydroxide, and calcium hydroxide [241]. The reaction is commonly carried out in the dark under an atmosphere of inert gas as N_2_, and ascorbic or ethylenedinitrilotetraacetic acid is generally used to prevent the degradation of compounds [68]. Similar to acid hydrolysis, alkaline conditions can lead to polyphenol degradation. In studies on quinoa, ferulic acid was released more efficiently by alkaline hydrolysis than by enzymatic treatment with pectinase. However, this method was associated with significant losses of ferulic acid and other polyphenols during processing [242]. Studies comparing acid and alkaline hydrolysis showed the higher efficiency of the former [243].

Enzyme-assisted hydrolysis enhances NEPs release through specific hydrolysis and requires complex enzyme manipulation for adequate hydrolysis [244]. Enzymes such as cellulases, hemicellulases, pectinases, amylases, and glucanases are used, either individually or in mixtures [241]. Apart from chemical hydrolysis, carbohydrate-hydrolysing enzymes can effectively release NEPs bound to sugar, fiber, or proteins inside the plant matrix [245]. This method allows the use of mild conditions under which most of the extractable compounds are stable. However, when referring to enzyme hydrolysis, the high cost of the enzyme and long processing time are the main hurdles for commercial applications [246].

The efficiency of hydrolysis methods described above (Table 8) could be enhanced using MAE, UAE, PLE methods. Since the application of these methods to the extraction of NEPs from various matrices has been described in a recent reviews by Ding et al. [67], Martins et al. [68] and especially Bhadange et al. [247], we will not discuss them in detail.

### 3.3. Purification of Extracts Containing Procyanidins

**Figure 15:**
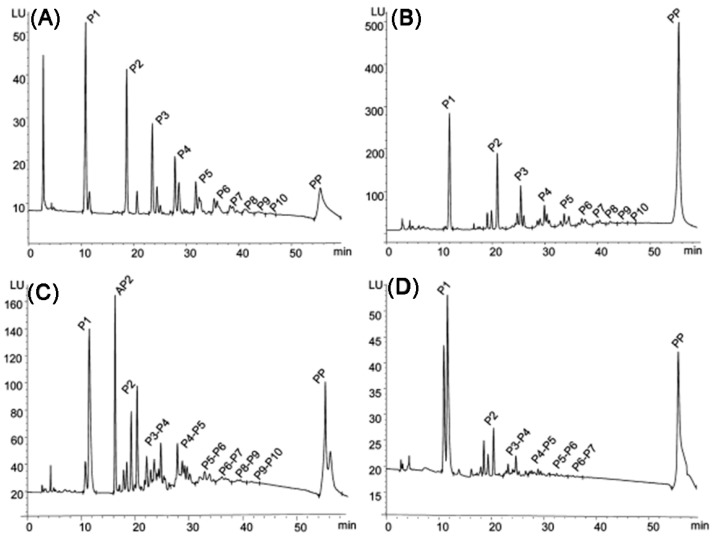
Normal-phase HPLC-FLD chromatograms trace of procyanidins from (**A**) apple, (**B**) pine bark powder, (**C**) lingonberry, and (**D**) grape. Labels P1–P10 indicate the degree of polymerization of procyanidins in the peaks. The single peak labeled AP2 indicates an A-type dimeric procyanidin. Polymeric procyanidins with degree of polymerization > 10 appeared as a single peak at the end of the chromatogram. Reprinted with permission from [254]. Copyright 2023 American Chemical Society.

The extracts obtained contained dozens (e.g., Morazzoni et al. determined 71 compounds after extraction and three-step filtration [157]) of different components (Figure 15). Nonetheless, in industry, extracts are often purified only from toxic and harmful substances by high and low degree of polymerization fractionation without isolating individual compounds [157,248]. The most common purification methods used in industry include column separation [7,249,250], membrane filtration [251], and high-performance counter current chromatography (HPCCC) [252,253]. All the above methods allow the separation fractions of oligomeric and polymeric polyphenols, as well as the separation of sugars from them. The isolation of individual procyanidins is performed by laboratory methods, among which HPLC/UPLC with various detection methods dominates.

The most common purification method, both in industry and laboratory practice, is chromatographic separation on a column packed with hydroxypropylated dextran gel (Sephadex LH-20). This approach enables efficient removal of sugars, pigments (such as chlorophyll), and most interfering phenolic compounds [7]. Depending on the target product, water, methanol, ethanol, acetone, or their mixtures can be used as eluents [255]. The main drawbacks of this technique are its low resolution and the inability to isolate PPCs due to their irreversible interaction with Sephadex LH-20 [256]. Nevertheless, it remains a practical method for isolating alkaloids, mixtures of procyanidins with degree of polymerization < 3, and high molecular weight oligomeric fractions.

For more refined molecular weight separation, preparative and semi-preparative columns based on C18 and C18-diol stationary phases can be employed. However, neither provides sufficient selectivity for isolating individual procyanidins [257]. A potential solution is to combine both chromatographic techniques to achieve better resolution

Another simple and selective method suitable for semi-industrial and industrial applications is membrane filtration. This technique relies on selective permeable membranes to separate oligomers by size. Gutierrez-Docio et al. demonstrated that a 10 kDa membrane can effectively separate OPCs with degree of polymerization < 5 from the polymer fraction [258]. Reig-Valor et al. compared ultrafiltration (UF) and nanofiltration (NF) systems for polyphenol recovery from wine lees at the pilot scale. It was shown that the UF membrane, operated at a constant pressure of 2 bar, exhibited a permeability of 8.2 ± 0.37 L/bar·m^2^·h, while the NF membrane achieved a permeability of 8.2 ± 0.24 L/bar·m^2^·h at a constant temperature of 20 °C. Cleaning tests demonstrated that achieving 90–100% membrane recovery required chemical treatment. Regarding the phenolic content after filtration, it was found that a 15 kDa pore-size UF membrane retained 54.2 ± 3.0% of the phenolic compounds in vinasse. Subsequent treatment of the permeate with a membrane allowing the passage of molecules smaller than 340 Da showed that 90.1 ± 1.1% of phenolic compounds were too large to pass through, leaving only a minor fraction in the filtrate. Introducing a UF pretreatment step before NF significantly reduced membrane fouling during NF and improved the overall extraction efficiency of target compounds [259]. The disadvantages include relatively low filtration rate as well as high capital cost due to the high cost of the membrane. Nevertheless, this technology is used for the separation and purification of procyanidins with different degree of polymerization on an industrial scale [200].

A third method suitable for industrial use is HPCCC. Unlike traditional column chromatography, HPCCC does not use solid stationary phases. Instead, it relies on the partitioning of solutes between two immiscible liquid phases (fixed and mobile) under complex hydrodynamic motion in rotating coil. The system reaches unidirectional hydrodynamic equilibrium, enabling effective separation [260]. Commonly used solvent systems include water, acetonitrile, methanol, ethyl acetate, n-butanol, and hexane [261]. HPCCC can be combined with conventional column chromatography to further increase the purity of isolated compounds [252]. The key advantage of HPCCC is its ability to isolate individual procyanidins in significant amounts. Previous studies have shown that HPCCC enables the purification of procyanidins B2, B3, B4, B5, B8, and C1 with 70–98% purity from various natural sources [14,262,263]. According to Li L. et al., this technique allows the isolation not only of dimeric but trimeric, tetrameric, and pentameric procyanidins, along with other polyphenolic compounds [253]. The main drawbacks of HPCCC are the relative complexity of the equipment and the long separation time.

In laboratory practice, more selective chromatographic methods are commonly used. The most widespread techniques for in vitro isolation of procyanidins are reversed- and normal-phase HPLC or UHPLC. Reversed-phase HPLC is well suited for isolating compounds with a low degree of polymerization 1–4, whereas normal-phase HPLC allows the isolation of compounds with degree of polymerization up to 10. For highly polar compounds, hydrophilic interaction liquid chromatography can be applied. However, this method is limited by relatively low column stability and long equilibration times. While laboratory chromatographic methods allow the isolation of almost all known low-molecular-weight procyanidins, it typically requires the use of multiple columns with different stationary phases, which complicates scale-up. Since 2000, numerous advanced chromatographic and mass spectrometric techniques have been developed [14], which are beyond the scope of this review.

For the analysis of PPCs with degree of polymerization > 4, pretreatment methods such as thiolysis [264] and phloroglucinolysis are commonly employed [265]. These reactions involve cleavage of the interflavan bond in oligomeric or polymeric polyphenols by a nucleophile (e.g., thiol or phloroglucinol). Under acidic conditions, the nucleophile reacts with the extension units of the polymer chain, forming nucleophile–monomer adducts, while the terminal units are released as flavan-3-ols [266]. Subsequent chromatographic analysis enables the separation of flavan-3-ols and adducts, allowing the estimation of the degree of polymerization. Although useful for qualitative screening and approximate PAC content determination, these methods do not provide detailed structural information. Additional modern laboratory techniques for the determination of procyanidins are discussed in several recent reviews [67,128,267].

Another promising method for the separation of various fractions of procyanidins involves the use of resins. Thus, Gutierrez-Docio et al. demonstrated that combining UF with solid-phase extraction on XAD7HP and XAD16 resins enables effective separation of polar compounds (sugars, sugar alcohols, di- and tricarboxylic acids) from OPCs. The UF step efficiently separated OPCs from PPCs, processing 7.2 L of clarified grape seed extract in under 3 h at a flux of 6–3.5 L/h·m^2^ and a pressure of 0.5 bar. SPE on XAD7HP and XAD16 effectively removed polar and ionic species, yielding a 9.6- and 7.8-fold enrichment of catechins and procyanidin dimers, respectively. However, resin adsorption capacity declined rapidly and irreversibly, likely due to OPC/PPC retention. This indicates that while such resins can be used for OPCs purification, their practical application is limited, and alternative adsorbents or resin regeneration strategies may be required [258]. Heravi et al. using a nonionic acrylic polymeric resin obtained more promising outcomes of extraction efficiency of 10.67% *w*/*w* in pilot plant [268].

Thus, the outcome can be summarized as follows: the main difficulty in obtaining procyanidins from plant raw materials is the purification of the resulting extracts. At the same time, the methods of laboratory purification of procyanidins are well studied and can be easily found in the literature, while industrial methods of purification are rather scarce, costly and deserve more attention from scientists and engineers.

### 3.4. Patents

Analysis of recent patent developments highlights growing interest in the practical applications of procyanidins beyond fundamental bioactivity research. A patent describes the development of oral formulations containing procyanidin rich plant extracts particularly from grape seeds, cocoa, and peanut skins for the prevention and treatment of endothelial inflammation and dysfunction, with specific relevance to COVID-19-related vascular damage [269]. This formulation provides clearly defined therapeutic use and dosing strategy, supporting the clinical potential of procyanidins as active pharmaceutical ingredients. In a related development, a strategy has been introduced to enhance the oral bioavailability of hydroxytyrosol through co-administration with selected polyphenols [270]. Although not limited to procyanidins, this patent underscores the synergistic potential of polyphenolic compounds to improve pharmacokinetic properties, offering valuable framework for the design of polyphenol-based therapeutics. Together, these patents reflect notable shift toward the targeted development of polyphenol-rich formulations with specific health benefits, bridging the gap between molecular understanding and practical biomedical applications.

### 3.5. Clinical Studies of Procyanidins

Growing evidence supports a causal role for flavanols in promoting cardiovascular health through the consumption of flavanol- or procyanidin-rich foods. However, the specific contribution of procyanidins themselves remains unclear, largely due to their poor absorption in the small intestine. It is hypothesized that procyanidins may exert cardiovascular effects indirectly by being broken down in the gastrointestinal tract into bioactive compounds. These breakdown products include flavanols possibly formed in the stomach and phenolic metabolites like 5-(3,4-dihydroxyphenyl)-γ-valerolactone produced by the gut microbiota. Confirming or disproving these mechanisms is crucial for accurately interpreting dietary studies and refining food composition databases [271]. A clinical trial assessed the effectiveness and safety of PCC1 and the senolytic compound *Cellumiva* in promoting skin rejuvenation over a 12-week period at oral dosage of 2.5 mg of procyanidin C1 was applied. Healthy women between the ages of 45 and 65 (74 participants), who were randomly assigned to receive either PCC1, Cellumiva (which contains procyanidin C1, Pterostilbene, and Spermidine), or a placebo were involved. Improvements in skin barrier function, wrinkle appearance, and overall skin texture and radiance were evaluated using imaging technologies and participant-reported outcomes [272]. Another trial was devoted to the evaluation of procyanidins in complex with chocolate; however, there are a number of other compounds that would have an effect, and it is complicated to find a clear correlation (Randomized Clinical Trial of the Effect of Cocoa Consumption in Cardiovascular and Immune Parameters in Colombian Patients With Newly Diagnosed Stage 1 Arterial Hypertension). The trials included adult participants aged 18–65 years (69 participants) with recently diagnosed (≤3 months) stage I essential arterial hypertension, for whom non-pharmacological management was indicated, and who voluntarily agreed to consume 50 g of chocolate daily for 12 weeks. Only flavanol monomers and, to a lesser extent, dimers are absorbed in the small intestine, while larger procyanidin oligomers are broken down by gut microbiota in the colon. This microbial catabolism produces metabolites particularly phenolic acids and valerolactones that enter circulation and peak 6–10 h after ingestion. The research aims to explore the time course of these metabolites and their potential impact on endothelial function following different patterns of procyanidin intake, an area not previously investigated [273]. Another clinical investigation focused on the in vitro effects of procyanidins on semen quality and sperm DNA fragmentation a prospective, double-blind study. This investigation aimed to determine whether the immediate addition of procyanidins to semen samples from infertile men influences standard semen parameters and sperm DNA fragmentation index. Overall, 50 male participants aged 18–50 years with infertility, defined as failure to achieve pregnancy after ≥12 months of unprotected intercourse (or ≥6 months when the female partner was >35 years old), were included. Data provides insight into the role of procyanidins in improving male reproductive health [274]. Additionally, effects of procyanidins on semen parameters and DNA fragmentation index during cryopreservation of abnormal human semen samples was studied. The research investigated whether supplementing semen samples from infertile men with procyanidins before cryopreservation outcomes in improved post-thaw semen parameters and a lower sperm DNA fragmentation index, compared to untreated samples. Using a prospective, double-blind design, the research aimed to evaluate the potential protective role of procyanidins during the freezing and thawing process of abnormal semen samples [274].

Grape seed extract ecovitis, characterized by low levels of monomeric catechins and a high concentration of OPCs and PPCs, may influence the gut microbiota composition in patients with ulcerative colitis during remission The key questions this exploration seeks to address are as follows: (a) does ecovitis improve the gut microbiota profile in ulcerative colitis patients?; (b) does it affect intestinal permeability?; (c) how does it impact patients’ quality of life? A total of 25 volunteers aged 18–80 years participated, and results are expected on 12 June 2026 [275].Grape seed proanthocyanidins have demonstrated a hypotriglyceridemic effect following both acute and chronic consumption in rodent studies. The impact of a single, acute intake of proanthocyanidins prior to breakfast on plasma triglyceride levels in humans was considered. Moreover, the investigation seeks to clarify the underlying mechanisms responsible for the triglyceride-lowering effects of grape seed proanthocyanidins and to assess their influence on vascular function [276].

Proanthocyanidins have been suggested as a promising adjunctive therapy for periodontal treatment due to their strong antibacterial and anti-inflammatory properties demonstrated in preclinical studies. Proanthocyanidins selectively target periodonto-pathogenic bacteria such as *Porphyromonas gingivalis* while preserving beneficial oral commensals like *Streptococcus salivarius.* This clinical trial enrolled patients with stage III-IV periodontitis who received either minimally invasive non-surgical therapy alone or the same therapy combined with subgingival application of collagen hydrogels containing proanthocyanidins. Clinical periodontal parameters including probing pocket depth, clinical attachment level, bleeding on probing, and plaque index were assessed before treatment and after 8 weeks, alongside measurements of salivary immunological markers MMP-3 and TIMP-1. Overall 46 participants with age of 30 years and older [277].

The Study of a Food Supplement (Mannose and Proanthocyanidins Prolonged Release 24 h) Versus Proanthocyanidins in the Prevention of Urinary Tract Infections in Kidney Transplant Patients was completed in 2023. The properties of D-mannose and proanthocyanidins make it worthwhile to investigate the clinical benefits of using dietary supplements containing both compounds to prevent urinary tract infections without relying on antibiotics A total of 60 participants aged ≥18 years, all of whom were recipients of a cadaveric donor kidney transplant, were included. Participants were randomly assigned in a 1:1 ratio to one of two treatment arms (30 patients per group), receiving either Manosar^®^ or proanthocyanidins [278]. Adding d-mannose to proanthocyanidins did not significantly reduce the incidence of urinary tract infections or asymptomatic bacteriuria compared with proanthocyanidins alone within the first 6 months after kidney transplantation. A total of 27% of participants experienced at least one UTI episode, asymptomatic bacteriuria was common (~57%), and there were no statistically significant differences between the treatment groups in UTI, cystitis, pyelonephritis, or bacterial species isolated [279].

### 3.6. Metabolism and Bioavailability of Proanthocyanidins

Procyanidins show limited intestinal absorption in their intact oligomeric forms, with monomers being partially absorbed in the small intestine, while higher oligomers predominantly reach the colon and undergo extensive microbial metabolism. The resulting low-molecular-weight phenolic metabolites, rather than native procyanidins, are absorbed, circulate at measurable systemic levels, and are considered the primary mediators of their biological effects [52]. Proanthocyanidins exhibit poor absorption in their native oligomeric forms, but are extensively metabolized by the gut microbiota Into low–molecular-weight phenolic acids and valerolactone derivatives, which are readily absorbed and detected in plasma mainly as glucuronide and sulfate conjugates at low micromolar concentrations. These circulating metabolites have been shown to exert biologically relevant antioxidant, anti-inflammatory, vasoprotective, and antimicrobial effects, suggesting that the systemic health benefits of proanthocyanidins are largely mediated by their microbial metabolites rather than the parent compounds [43]. Most orally ingested oligomeric and polymeric proanthocyanidins reach the colon, where a minor fraction is metabolized into phenolic acids and valerolactones, while the remainder modulates gut microbiota composition. This modulation includes enhanced microbial diversity, enrichment of beneficial taxa (e.g., *Akkermansia muciniphila*), increased butyrate-producing bacteria, and reduced LPS-producing microbes, collectively contributing to improved metabolic health and protection against metabolic and neurodegenerative disorders [280]. Measurements were taken at an approximate polyphenol intake of 1 g/day, although this value remains uncertain due to incomplete compositional data for several major polyphenol classes in foods. Human bioavailability studies indicate that plasma concentrations of individual phenolic compounds rarely exceed 1 µM after intake of 10–100 mg, while overall circulating phenol levels are likely higher due to the presence of tissue- and microbiota-derived metabolites [281].

In vitro incubation of purified proanthocyanidin polymers with human colonic microflora under anoxic conditions demonstrated near-complete degradation within 48 h, yielding phenylacetic, phenylpropionic, and phenylvaleric acids with meta- or para-hydroxylation patterns similar to those produced from flavonoid monomers. These findings provide the first clear evidence that dietary proanthocyanidin polymers are converted into low-molecular-weight aromatic metabolites, highlighting the importance of considering the biological activity of these metabolites when evaluating the nutritional effects of proanthocyanidins [282].

Thus, the most recent review by Espín et al. stated about inter-individual variability in clinical responses to proanthocyanidins is likely driven by differences in gut microbiota composition and metabotypes, underscoring the need for future stratified clinical trials to distinguish the biological roles of the parent compounds, microbial communities, and specific microbial metabolites [283].

## 4. Challenges and Future Outlook

In the last decade, procyanidins have attracted the close attention of both synthetic chemists and scientists involved in the learning of bioactive compounds. Despite the fact that the biological activity of these compounds is well studied, the mechanisms behind the manifestation of certain properties are not fully known. To estimate the fundamental mechanisms responsible for the manifestation of certain properties, methods are needed that allow obtaining procyanidins in significant quantities. One of the solutions is to obtain extracts of natural raw materials and purify them. And although modern extraction methods make it possible to extract significant amounts of target substances, effective methods for isolating individual compounds from multicomponent extracts have not yet been developed.

An alternative is the direct synthesis of target compounds. There are several fundamentally different approaches to the preparation of OPCs: these are direct intermolecular condensation methods and intramolecular condensation methods. The most versatile option is the orthogonal approach, which makes it possible to stereoselectively obtain OPCs with different degrees of polymerization. However, some procyanidins are not available for synthetic methods of preparation.

Knowledge about the conformational features of most of the main procyanidins necessary for constructing theoretical models was obtained at the beginning of the XXI century. Currently, the structures of several undescribed compounds are being refined using the latest methods of quantum chemical modeling. Based on this data and in vitro experiments, a number of models were built to explain the activity of procyanidins, for example, antioxidant activity or the principles of interaction with proline-rich peptides. However, when moving to in vivo studies, these models in some cases lose their predictive ability. Solving this problem and constructing working models that relate the structural aspects of procyanidins and their properties requires the collection of more data and taking into account a significant number of various factors.

Thus, we identified several challenging directions

- Development of effective methods for deep purification of multicomponent extracts and for isolating individual procyanidins from natural raw materials.
- Development of synthetic regioselective methods for obtaining procyanidins B5, B7 and B8 and other analogs not yet synthesized.
- Development and construction of predictive models that could work both in vitro and in vivo.

The solution of abovementioned problems will not only allow evaluations of the structure–property correlations for these compounds, but also pave the way for the production/synthesis of these compounds on a sufficient scale.

## Figures and Tables

**Table 1:** Theoretical and experimental content of various isomers of procyanidins B1-B4 in aqueous solutions [54].

Procyanidin Type	Conformation (C-F Rings)	Eq-Eq, %	Eq-Ax, %	Ax-Eq, %	Sum, %
**B1**	compact (exp.)	83.7	5.7	2.6	92.0
	compact (theor.)	96.7	0.2	1.9	98.8
	extended (exp.)	7.3	0.5	0.2	8.0
	extended (theor.)	0.4	0.0	0.8	1.2
**B2**	compact (exp.)	53.4	0.0	1.6	55.0
	compact (theor.)	96.2	0.3	1.6	98.1
	extended (exp.)	43.7	0.0	1.3	45.0
	extended (theor.)	0.95	0.05	0.9	1.9
**B3**	compact (exp.)	75.0	14.3	4.7	95.0
	compact (theor.)	98.2	0.2	0.9	99.3
	extended (exp.)	4.0	0.8	0.2	5.0
	extended (theor.)	0.7	0.0	0.0	0.7
**B4**	compact (exp.)	70.0	9.0	4.0	83.0
	compact (theor.)	96.5	1.1	0.6	98.2
	extended (exp.)	14.0	1.0	2.0	17.0
	extended (theor.)	1.7	0.1	0.0	1.8

**Table 2:** Surface areas and energies of the ground states of procyanidins [82,85].

Procyanidin Type and Conformation	Surface Area, Å^2^	ΔE_water_, kcal/mol
A1	710.0	0.8
A2	725.0	0.0
B1 extended	725.0	4.86
B1 compact	709.0	0.0
B2 extended	726.0	0.8
B2 compact	699.0	0.0
B3 extended	745.9	6.49
B3 compact	678.5	3.49
B4 extended	744.4	0.23
B4 compact	677.3	0.0
B5	748.3	5.05
B6	751.4	3.12
B7	745.3	4.63
B8	749.8	4.55
C1 compact-compact	979.0	1.38
C1 compact-extended	-	1.89
C1 extended-compact	1038.0	0.0
C1 extended-extended	-	1.9.0

**Table 3:** Major synthetic strategies of various procyanidins.

Procyanidin Type	Synthesis Type	Ref
A1, A2	Radical or fermentative oxidation of B1 or B2 procyanidins	[115,116,129,130,131]
	Intermolecular condensation of catechin blocks	[115,116,132,133]
	Synthesis using non-catechin precursors (diphenyl-propene derivatives)	[116]
B1-B4	Intermolecular condensation of catechin blocks (nucleophilic and electrophilic) in the presence of Lewis acids	[120,122,134,135,136]
	Intramolecular condensation of a molecule with a glutar or amber bridge	[137]
	Orthogonal synthesis	[122]
B5-B8	Intermolecular condensation of catechin blocks (nucleophilic and electrophilic) in the presence of Lewis acids	[120,138]
	Intramolecular condensation of a molecule with an azelaine bridge	[123]
	Orthogonal synthesis	[122]
C1, C2	Intermolecular condensation of B-type procyanidins and catechin blocks in the presence of Lewis acids with an excess of a nucleophilic agent	[119,139,140]
	Orthogonal synthesis	[122]

**Table 4:** The procyanidins content in various wastes of the agro-industrial complex.

Plant	Waste Type	Procyanidin Type	Concentration	Ref
Lychee (*Litchi chinensis*)	Leaves	A2	44.8–69.6 mg/g	[6]
Common grape vine (*Vitis vinifera* L.)	Pomace	Dimeric procyanidins	1.0–3.0 mg/g	[153]
		Total polymeric procyanidins	27.0–43.3 mg/g	
Saskatoon berry (*Amelanchier alnifolia*)	Peel and seeds	A-type dimers	0.2–0.46 mg/g	[154]
		B-type dimers	0.026–0.06 mg/g	
		Polymeric procyanidins	14.5–26.6 mg/g	
Cacao (*Theobroma cacao* L.)	Husk	B1	0.55–0.83 mg/g	[13]
		B2	0.23–0.90 mg/g	
Nepali hog plum (*Choerospondias axillaris*)	Peel	Total procyanidins	42.8–120.0 mg/g	[155]
Monterey pine (*Pinus radiata*)	Bark	Total procyanidins	404.0 mg/g	[156]
Pitch pine (*Pinus rigida*)			489.0 mg/g	

**Table 5:** Selected methods for procyanidins obtaining from plant materials using combined extraction methods.

Part of a Plant	Raw Material	Preliminary Preparation	Extraction	Purification	Ref.
Bark	Young shoots of hawthorn (*Crataegus monogyna* Jacq.)	Washed with 80% acetone; stored for 24 h at 18 °C; sonicated for 30 min at −20 °C	Water and chloroform (ratio not established)	1. NaCl solutions; 2. Acetate water 0.5:5 (*v*/*v*) 3. Chloroform and hexane	[174]
	Maritime pine *(Pinus pinaster* Ait.)	Grinding, screening through 60 and 18 mesh	CO_2_-ethanol (90:10), 3 times, 323 and 303 °C, 20.3 and 25.1 MPa, 370 and 360 min; 7.6, 13.2, and 19.1 kg/s × 10^5^, solvent-to-solid mass ratio 28:1, 2:1, 20:1	Hydrodistillation in a Schilcher apparatus, following the AOAC 962.17 method	[186]
Flowers	Small-leaved linden (*Tilia cordata* Mill.)	Dry in the dark; fine grind	Acetone methanol water (3:1:1, *v*/*v*/*v*); exhaustive ultrasonic-assisted extraction 5 times for 30 min	Column chromatography using Sephadex LH-20; Column chromatography using Toyopearl HW-40F; Column chromatography using Diaion HP-20	[170]
Fruits	Lychee pericarp (*Litchi chinensis* Sonn.)	Mix with chilled aqueous ethanol for 3 min and then homogenize in ice bath at 10,000 rpm for 5 min	Ultra-high-pressure extraction pressure 295 MPa, holding time under pressure 13 min, liquid to solid ratio 16:1, 70% ethanol, 25 °C	Information is not presented	[180]
	Apples (*Malus domestica*)	(Not described)	Supercritical fluid, sublimation	HPLC followed by polyamide MN column	[11]
	Grape pericarp (*Vitis vinifera* L.)	Freezed in liquid nitrogen; grinded with a pestle in a mortar	Acetone/water (70 30, *v*/*v*) containing 0.05% (*v*/*v*) trifluoroacetic acid Stir for 1 h at room temperature in the dark	Column chromatography using Toyopearl TSK-HW 50F	[143]
	Grape pomace (*V. vinifera* L. cv. ‘Tempranillo’)	Frozen at 80 °C, freeze-dried under vacuum and milled	Pressurized hot water extraction 2 cycles; 10 min; 1500 psi. Choline chloride/oxalic acid (1:1, *v*/*v*) 30% mixture in water	Extracts were filtered through 0.20 μm polyester filters, then separated on an Ascentis Express C18 (HPLC)	[188]
	Avocado (*Persea americana* Mill.)	Washed; sublimated; grinded	Ultrasonic-assisted extraction three times with a solution of acetone/water (70:30, *v*/*v*) at 25 °C; add 0.1% ascorbic acid	Column chromatography using Sephadex LH-20 (50% MeOH/H_2_O and 70% Acetone/H_2_O)	[7]
Seeds	Peanut peel (*Arachis hypogaea* cv. Runner 886)	Lyophilization	Acetone water (60:40), pH 1.5; 70 °C; 30 min	Amberlite XAD-2; Sephadex LH-20; TCX	[166]
	Cacao beans (*Theobroma cacao* L.)	Washed; frozen; (liquid nitrogen); ground	Extracted three times with n-hexane (solid/liquid 1:5, *w*/*v*), 80% aqueous methanol (*v*/*v*) and 75% aqueous acetone (*v*/*v*), evaporated at 30 °C, extraction with an equal volume of chloroform three times	High performance counter current chromatography (HPCCC)	[14]
	Blackberry (*Rubus* spp.)	Pressed to obtain pulp, the pulp was roughly homogenized	Extracted by distilled water, methanol and ethanol (conventional solvents) or Natural deep eutectic solvents in a sonication water bath. An amount of 0.5 g was mixed with 10 mL of conventional or Natural deep eutectic solvents. The mixture was ultrasonicated at 25 °C for 20 min. Filtered through Whatman filter paper No.1 three times.	HPLC using Inertsil ODS-4	[187]
	Iris lactea (*Iris lactea* Pall. *var. Chinensis* (Fisch.))	Air dry; grinded to powder	Supercritical extraction with carbon dioxide; residue 80% ethanol (3 × 25 L, every 3 h) at 60 °C	Column chromatography using silicagel; HPCCC	[183]
	Grape seeds (*Vitis vinifera* L.)	The samples were processed to granulometry of 2 mm	ultrasound bath (Water ethanol (3:7 *v*/*v*), 34 kHz), vacuum rotary evaporation (35 °C), and wash by n-hexane, vacuum rotary evaporation.	A Sephadex LH-20 column rinse sequentially by ethanol/water 80:20 (*v*/*v*) and ethanol/water 50:50 (*v*/*v*),	[190]
	Black soy husk (*Glycine max*)	Stored at 10 °C before use	70% acetone/0.5% acetic acid solution for 3 h at room temperature	Column chromatography using Sepabeads SP700; reverse phase preparative HPLC	[169]

**Table 6:** The main advantages and disadvantages of methods using for procyanidins extraction.

Extraction Technic	Advantages	Disadvantages
Classical extraction	Easy Very low costs	Long time of extraction (hours) Low efficiency
UAE	Medium time of extraction (dozens of minutes) Low costs	Medium efficiency
MAE	Short time of extraction (minutes) High efficiency Low solvent consumption	High costs The impossibility of extracting heat-sensitive components
Supercritical CO_2_	Maximal efficiency	Very high costs
PLE	High efficiency Low solvent consumption	High costs
DES assisted extraction	Short time of extraction (minutes) Low solvent consumption Great potential of combination with other technics	Questionable results

**Table 7:** NEPs extractions and main results.

Source	NEPs Release Method	Main Compounds	References
Apple pomace	Sequence extraction combining acid and alkaline hydrolysis	The highest total phenolic content, reaching 12.38–13.76 mg GAE/g dry weight, with quercetin-3-O-galactoside identified as the predominant compound (by MS).	[229]
	Sequential extraction facilitated by microwave	Flavan-3-ols constitute the major polyphenolic class (2.88 g/kg dry weight) with an average degree of polymerization of 4.7. Alkaline hydrolysis of apple procyanidins generates 3,4-dihydroxybenzoic acid (0.67 M/kg) and catechol (0.15 M/kg) as the principal degradation products.	[178]
Grape pomace	Enzyme hydrolysis	Under optimized conditions the total phenolic content reaches 0.81 g GAE/100 g dry weight, with gallic acid (0.16 g/100 g dry weight) emerging as the main hydrolysis product.	[230]
Roselle by-products	Acid hydrolysis	The contents of hydrolysable polyphenols and proanthocyanidins are 6.18 mg GAE/g and 6.67 mg proanthocyanidin eq/g, respectively. Notably, NEPs account for 71.2% of the total phenolic content.	[69]
Brown rice bran	Alkaline hydrolysis	The total phenolic content reaches 276 mg GAE/100 g dry weight. Ferulic acid (1617 μg/g dry weight) and p-coumaric acid (394 μg/g dry weight) are the main compounds after hydrolysis.	[235]

**Table 8:** Summary of the different approaches for NEPs extraction.

NEPs Release Methods	Advantages	Disadvantages	Improvements
Acid hydrolysis	Cleavage of glycosidic bonds in the cell wall	Waste generation. High temperatures and acid concentrations	Milder conditions
Alkaline hydrolysis	Break ester bonds in the cell walls, releasing phenolic polysaccharide compounds	Waste generation. Complex pre-treatment process Inert gas atmosphere to prevent NEPs oxidation	Milder conditions
Enzymatic hydrolysis	Fast Low temperature Green technology	High cost Low specificity	Further studies about the methodology and recovery of enzymes
Additional extraction technologies	Break bonds with mechanical actions, increasing the solubility Increasing the efficiency of the extraction Reducing time Green technology	High cost Specific equipment	Further studies about the methodology

## Data Availability

No new data were created or analyzed in this study. Data sharing is not applicable to this article.
